# Drop‐Location‐Insensitive Droplet‐Based Electricity Generator Using Polydimethylsiloxane Slippery Covalently Attached Liquid Coatings

**DOI:** 10.1002/advs.76947

**Published:** 2026-08-05

**Authors:** Jung Bin Yang, Donghyeon Yoo, Hideaki Teshima, Donghan Lee, Dongwhi Choi, Dong Rip Kim, Nenad Miljkovic

**Affiliations:** ^1^ Department of Mechanical Science and Engineering University of Illinois at Urbana‐Champaign Urbana Illinois USA; ^2^ School of Mechanical Engineering Hanyang University Seoul South Korea; ^3^ International Institute for Carbon‐Neutral Energy Research (WPI‐I2CNER) Kyushu University Nishi‐ku Fukuoka Japan; ^4^ Department of Aeronautics and Astronautics Kyushu University Nishi‐ku Fukuoka Japan; ^5^ Department of Mechanical Engineering (Integrated Engineering Program) Kyung Hee University Yongin Gyeonggi Republic of Korea; ^6^ Materials Research Laboratory University of Illinois at Urbana‐Champaign Urbana Illinois USA; ^7^ Department of Electrical and Computer Engineering University of Illinois at Urbana‐Champaign Urbana Illinois USA; ^8^ Institute For Sustainability Energy and Environment (iSEE) University of Illinois Urbana Illinois USA; ^9^ Air Conditioning and Refrigeration Center University of Illinois Urbana Illinois USA

**Keywords:** contact electrification, droplet‐based electricity generator, energy harvesting, slippery surface, triboelectric nanogenerators

## Abstract

Droplet‐based electricity generators (DEGs) offer a promising strategy for harvesting untapped water energy from sources such as rain. To ensure high energy harvesting performance, droplets must contact the surface and be rapidly detached. To enable this behavior, superhydrophobic surfaces have been considered a solution. However, conventional superhydrophobic‐DEGs often face difficulties in practical applications due to non‐uniform performance caused by droplet bouncing and splitting. Here, we introduce a polydimethylsiloxane slippery covalently attached liquid (PDMS‐SCAL) coating into a DEG platform to minimize contact line pinning and droplet rebound. The PDMS‐SCAL‐DEG exhibits high open‐circuit voltage output (162.2 ± 3.8 V) and excellent performance stability across various droplet heights, incline angles, and drop locations. In drop‐location sensitivity experiments performed 14.1 mm apart from the optimum point, only a ≤25% performance degradation was observed. Experiments on a superhydrophobic‐DEG revealed that the same conditions led to a more than 92% performance degradation. Developed PDMS‐SCAL‐DEG maintains stable energy‐harvesting performance even after 10000 energy‐harvesting cycles. The reliability of PDMS‐SCAL‐DEG was also demonstrated through field testing in rainy environments. We further confirmed the comprehensive durability, including mechanical and chemical durability. This work presents a previously unexplored DEG platform with drop‐location‐insensitive characteristics to expand the scope of DEG applications.

## Graphical abstract

Drop‐location sensitivity remains a key barrier to practical droplet‐based electricity generators (DEGs). Here, a polydimethylsiloxane slippery covalently attached liquid (PDMS‐SCAL)‐coated interface promotes droplet sliding instead of splitting or bouncing, allowing stable electricity generation from randomly falling raindrops. The resulting PDMS‐SCAL‐DEG combines robust output, durability under harsh conditions, transparency, and flexibility, advancing practical water‐energy harvesting technologies and applications.

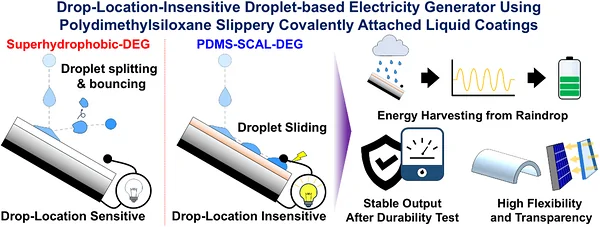

## 1 Introduction

The growing global need for renewable and sustainable energy has driven significant interest in innovative energy harvesting methods [1, 2, 3]. Covering 70% of the Earth's surface, water provides an immense yet underutilized energy source through its kinetic and potential energy, found in ocean waves, raindrops, and rivers [4, 5]. For the application of such unused water energy sources, droplet‐based triboelectric nanogenerators (TENGs) have been proposed, which can harvest energy by the principles of contact electrification and electrostatic induction between water droplets and dielectric insulating materials [6, 7]. Despite their potential, droplet‐based TENGs suffer from low power output, limiting their practical applications [5, 6]. To overcome this limitation, Xu et al. [8] first proposed a droplet‐based electricity generator (DEG) consisting of a dielectric layer with electrodes on both top and bottom surfaces (upper and bottom electrodes). The TENG and DEG systems share the same fundamental processes of liquid‐solid contact electrification and electrostatic induction, but they differ in the charge‐transfer pathway. TENGs rely on the combination of contact electrification and electrostatic induction using only lower electrodes. Sequential droplet impacts deposit surface charges on the dielectric layer, creating an electric field that induces charges in the lower electrode. When subsequent droplets slide over the dielectric layer, this electric field is temporarily screened, weakening the force holding the induced charges and causing an induced current to flow. In contrast, DEG introduces an upper electrode that fundamentally alters the electrical circuit. When a droplet bridges the dielectric and the upper electrode, it directly participates in the charge‐transfer process. As demonstrated by Xu et al. [8] via molecular dynamics simulations, the droplet's direct contact triggers intense polarization of positive and negative ions at the interface. This screening effect and subsequent droplet detachment induce a massive amount of charge transfer that cannot be achieved through lower electrodes alone. This mechanistic difference leads to a drastic increase in power output. As a result, DEGs can generate power output several orders of magnitude higher than conventional droplet‐based TENGs [5, 8].

Based on the enhanced power output of DEGs, many researchers have focused on their applicability [4, 5, 6, 8, 9, 10]. The need to broaden the droplet volume range that operates has arisen from consideration of microscopic water cycles such as fog, precipitation, and dew [4]. As the droplet volume decreases, alternative methods to separate them from the surface are required, given their reduced gravitational influence. In this regard, a superhydrophobic surface with low surface energy and high surface porosity is preferred. It enables droplets to easily roll off the DEG surface without being pinned, allowing energy harvesting from droplets with volumes of several microliters [4, 9]. However, the superhydrophobic surface has a low contact area with droplets, and high velocity droplets often rebound or break apart on its surface during impact. This phenomenon interferes with contact electrification and electrostatic induction, thereby reducing the power output of the DEG [11, 12, 13]. Specifically, when impacting droplets splash or fragment, the original droplet is divided into smaller satellite droplets, which reduces the effective liquid‐solid contact area and interrupts continuous charge transfer during the spreading process [14, 15, 16, 17]. In DEG operation, this effect is particularly detrimental because the droplet must maintain sufficient contact with both the dielectric surface and the upper electrode to form a stable discharge pathway. Therefore, suppressing satellite droplet formation is essential for maintaining efficient and reproducible droplet‐based electricity generation under practical impact conditions. In addition, this narrows the optimal droplet drop location for maximizing power output on the DEG surface, significantly limiting the broadening of DEG applications [18, 19, 20, 21]. Slippery lubricant‐infused porous surfaces (SLIPS) can serve as an alternative to superhydrophobic surfaces to minimize droplet breakup and splashing [21]. However, lubricant depletion remains a significant challenge on SLIPS surfaces [22, 23, 24].

Recently, a slippery covalently attached liquid (SCAL) coating has been introduced to overcome the lubricant depletion problem of SLIPS for a variety of applications [25, 26]. SCAL coating consists of a polymer brush covalently bonded to the surface, with the other end capable of freely rotating, bending, and stretching [27, 28, 29]. Its solid‐lubricated polymer brushes can significantly improve stability by eliminating lubricant depletion due to evaporation or washing by strong covalent bonding [27, 30]. In addition, these polymer brushes' long bond length and large bond angle (here, we used a polydimethylsiloxane (PDMS) brush) impart slippery properties to the surface (usually a water droplet sliding angle of less than 5°) [27, 28]. In this study, we first introduced a novel DEG platform enhanced with a PDMS‐based SCAL coating, providing stable and efficient electricity generation (Figure 1a). The novelty of this work doesn't lie in the concept of a “slippery surface” itself. The core distinction of our study is that, while existing DEGs have primarily focused on maximizing their performance at optimal droplet impact locations, we identified “drop‐location sensitivity” a critical bottleneck in actual rainfall environments, as the key challenge. To address this, we introduced a lubricant‐free, fluorine‐free, covalently attached, liquid‐like PDMS‐SCAL interface into our DEG system. In particular, the PDMS‐SCAL‐DEG significantly mitigated the drop in power output observed during off‐optimal impacts compared to superhydrophobic‐DEGs. Furthermore, we validated these advantages from an application‐oriented perspective (Figure 1b) through droplet hydrodynamics analysis, field validation under actual rain and roof‐drip conditions, and assessments of mechanical, chemical, and optical durability. Consequently, the novelty of this research lies not merely in the surface modification but in the proposal of a robustness‐centered design strategy for practical, real‐world DEG applications.

**FIGURE 1:**
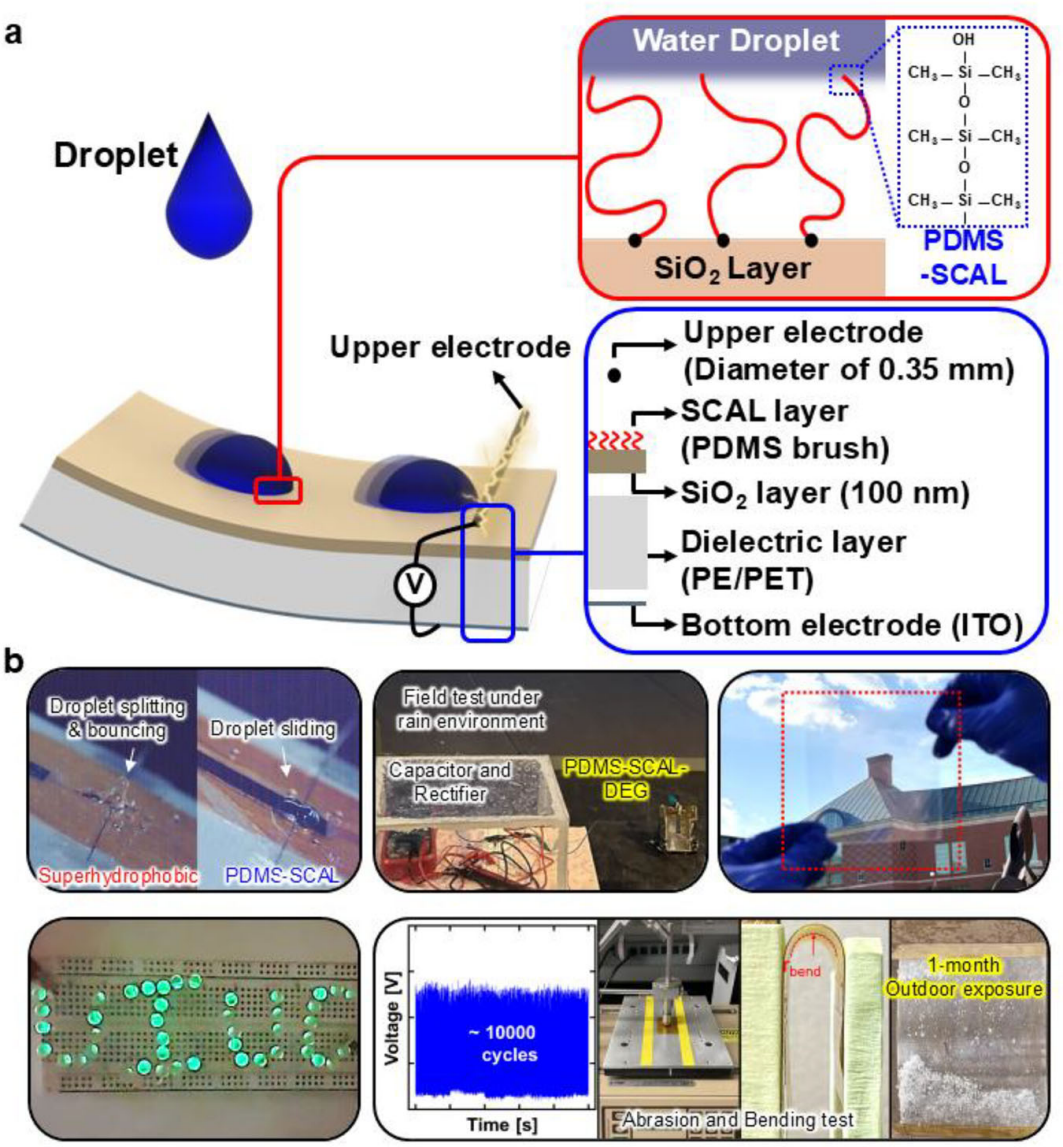
Schematic illustration of the structure of the polydimethylsiloxane slippery covalently attached liquid droplet‐based electricity generator (PDMS‐SCAL‐DEG) and its features. (a) PDMS‐SCAL‐DEG consists of an upper electrode, PDMS‐SCAL coating layer, SiO_2_ intermediate layer, dielectric layer (PE/PET), and bottom electrode. (b) PDMS‐SCAL‐DEG's novelty: drop‐location‐insensitivity, field test under actual rain environment, transparency for enhancing the applicability, relatively high performance without any fluorinated materials, and comprehensive durability test. Schematics are not to scale.

## 2 Results and Discussion

### 2.1 Surface Characteristics and Drop‐Location‐Insensitivity Performance of Superhydrophobic‐ and PDMS‐SCAL‐DEG

The DEG performance was characterized by using a commercial PET‐PE dielectric film coated with ITO electrodes (square resistance of 100Ω/sq). ITO, PET, and PE layers have thicknesses of 1.9 ± 0.1, 50.6 ± 2.6, and 149.0 ± 5.0 µm, respectively, as shown in Figure S1 (Supporting Information). The surface characteristics of the superhydrophobic‐DEG are shown in Figure S2. In this study, AF2400 resin, a solvent‐soluble PTFE, was used as a binder and formed a surface with high roughness (159.7 ± 43.7 nm) using PTFE NPs (Figure S2a,b). The fabricated superhydrophobic‐DEG surface was confirmed to have excellent superhydrophobic properties (receding apparent contact angle of 152.1 ± 1.7° and contact angle hysteresis (CAH) of 5.0 ± 2.0°) even at a low coating thickness (1.67 ± 0.15 µm), as shown in Figure S2c,d. The excellent water repellency characteristics of superhydrophobic‐DEG surfaces facilitate rapid droplet removal from the surface after energy generation through expansion and contraction when droplets fall onto it. The chemical composition of Superhydrophobic‐DEG is shown in Figure S2e. Specifically, the atomic percentages of C, O, and F elements are approximately 31.1%, 12.4%, and 56.5%, respectively. The superhydrophobic‐DEG exhibits excellent voltage output (236.8 ± 4.6 V), as shown in Figure S2f,g (surface inclined angle of 45° and droplet height of 25 cm). Figure S2h shows the amount of energy output (*E*_out_) corresponding to each moment of superhydrophobic‐DEG operation.

The enhanced energy generation of superhydrophobic surfaces is expected when considering the operation mechanisms of the DEG and its equivalent circuit. When surface charges are generated through contact electrification, counter‐ions or dipoles within the liquid droplet align accordingly. Those surface charges, along with the alignment, form an electrical double layer (EDL). Because these counter‐ions or dipoles are bound to the surface charges, the EDL stores charges. Therefore, the DEG can be modeled as an equivalent *RC* circuit. In this model, *R*_D_ is primarily determined by the resistivity of the liquid droplet, and *C* includes three capacitors in series: liquid droplet‐dielectric interface (*C*_EDL,1_), the liquid droplet‐upper electrode interface (*C*_EDL,2_), and liquid droplet‐dielectric layer‐ITO electrode (*C*_Bulk_) [8]. Figure S3 illustrates the model of the three capacitors during DEG operation. After sequential contact and separation of multiple droplets, the DEG dielectric film becomes negatively charged. The upper and bottom electrodes carry positive charges to maintain electrical equilibrium (Figure S2g‐(1)). When the next droplet comes into contact, the dipoles or ions of the droplet will be arranged because of the negatively charged dielectric film of the DEG. As a result, the equilibrium is disrupted, and some positive charges move from the bottom electrode to the upper electrode (Video S1). Consequently, *C*_EDL,1_ and *C*_Bulk_ are formed as shown in Figure S3‐(i). At this stage, the circuit remains open, and the two capacitors act as reservoirs for the surface charges generated by contact electrification. This charge's movement continues as the droplet spreads and the contact area increases. When the droplet reaches its maximum spreading area, the charge moves the most. Simultaneously, the droplet touches the upper electrode, triggering peak voltage output. As *C*_EDL,2_ is formed, the three capacitors and resistors constitute a closed circuit as shown in Figure S3‐(ii). In this state, stored charges move toward *C*_EDL,2_ due to the potential difference. The output depends on the drop location for the droplet and is the highest when the droplet touches the upper electrode with maximum spreading (Figure S2g‐(2)). After maximum spreading, the droplet begins to shrink, reducing its contact area with DEG. The effect of the droplet on the dielectric film's negative charges diminishes, resulting in the reversal of positive charge motion (from the upper electrode to the bottom electrode, Figure S2g‐(3)) as shown in Figure S3‐(iii). When the droplet completely detaches from the DEG, a cycle for energy generation for the DEG is complete, and the equivalent circuit returns to an open state (Figure S2g‐(4)). It is known that the open‐circuit voltage output increases as the rate of change of the droplet‐surface contact area during spreading decreases, and superhydrophobic surfaces are preferred to reduce this rate [4, 20]. When the droplet completely detaches from the superhydrophobic‐DEG, the energy‐generating cycle for the superhydrophobic‐DEG is completed (Figure S2g‐(4) and Figure S3‐(iv)). If there are residual droplets, the dipoles or ions within them interact with the negative charges on the dielectric film. Then, the movement of positive charges between the upper and bottom electrodes is delayed, resulting in degradation of the open‐circuit voltage output. To support this, we also measured surface potential changes in PDMS‐SCAL‐DEG, as shown in Figure S4. While directly observing dipole or ion movement is challenging, the movement of positive charges in the electrodes depends on the surface potential of the dielectric layer. We measured the potential of PDMS‐SCAL‐DEG under three conditions: pristine (initial), charged via contact electrification, and after the deposition of residual droplets. To generate these residual droplets, we intentionally altered the droplet release point to ensure they split upon the PDMS‐SCAL‐DEG surface. Surface potential was measured using a non‐contacting electrostatic voltmeter (Trek 347, Trek) with the probe positioned 3 mm above the surface. Measurements were taken across a 4 × 5 grid with 1 cm spacing along both the x‐axis and y‐axis. The experimental parameters, droplet release height, inclination angle, volume, and the number of falling droplets, were fixed at 25 cm, 45°, 49 µL, and 100, respectively. While the PDMS‐SCAL‐DEG was initially slightly negative, it became strongly negatively charged after contact electrification, as expected. However, the deposition of residual droplets shifted the surface potential toward a more neutral state. Given that only the residual droplets interacted with the PDMS‐SCAL‐DEG surface, we believe these results indirectly demonstrate that the residual droplets hinder the movement of positive charges, thereby degrading open‐circuit voltage output.

The water repellency and roughness of superhydrophobic surfaces trap air within the surface microstructures, which can be compressed by the impact of a water droplet, causing the liquid to bounce and split [13, 31, 32, 33]. Depending on the drop location of the water droplet, droplet bouncing can cause it to jump over the upper electrode. This limits the contact between the droplet and the surface and significantly reduces the energy harvesting performance [18, 19, 20]. Meanwhile, droplet fragmentation occurs when the kinetic energy of the droplet is converted into surface energy to form small satellite droplets, which split from the initial droplet during impact and spreading [34]. This behavior can be further understood from the role of texture‐confined air during droplet impact. Zhang et al. [33] compared droplet impact and rebound behavior on open‐ and closed‐cell superhydrophobic surfaces. They showed that air stored in closed microcavities prevents liquid penetration and functions as a “gas spring” that lifts the liquid lamella, enabling rebound even under high‐Weber‐number impact. Similarly, in the case of superhydrophobic‐DEG, micro‐ and nanoscale structures can retain air pockets. During droplet impact, these confined air pockets can be compressed and act as a gas spring, and during the release process, they promote lamella lifting and rebound. Once the spreading lamella becomes thin, roughness‐induced perturbations and upward recoil can destabilize the rim and locally rupture the liquid film [35]. The ruptured lamella then retracts due to surface tension and breaks into satellite droplets, thereby generating additional liquid‐air interfacial area. This phenomenon reduces the contact area between the droplet and the surface at the triple contact between the water droplet, the upper electrode, and the DEG surface. Hence, droplet splitting is another factor that degrades energy harvesting performance.

Figure 2a shows the uniformity of the energy generation of the superhydrophobic‐DEG surface with a droplet volume of 49 𝜇L, surface incline angle of *θ* = 15°–60°, and droplet initial height of *h* = 5–25 cm. To quantify the uniformity of the open‐circuit voltage output under droplet impact conditions, we derived the coefficient of variation (*CV*) value using Equation (3). A low *CV* value indicates uniform open‐circuit voltage output, while a high *CV* value indicates non‐uniform output. For example, the cases are compared where the water droplets were dropped on the superhydrophobic‐DEG surface at *θ* = 15° and 60° with *h* = 25 cm and *h* = 5 cm. For *h* = 5 cm, the generated energy was uniform (both *θ* = 15° and 60°) with a *CV* value of 12 ± 6% and 5 ± 3%, respectively. However, for *h* = 25 cm, a non‐uniform voltage distribution was observed at *θ* = 15° with a *CV* value of 59 ± 24%. This indicated that at lower inclination angles, it is easier for the droplet to split and bounce due to the greater inertial force component normal to the surface after impact, resulting in higher *CV* [36].

**FIGURE 2:**
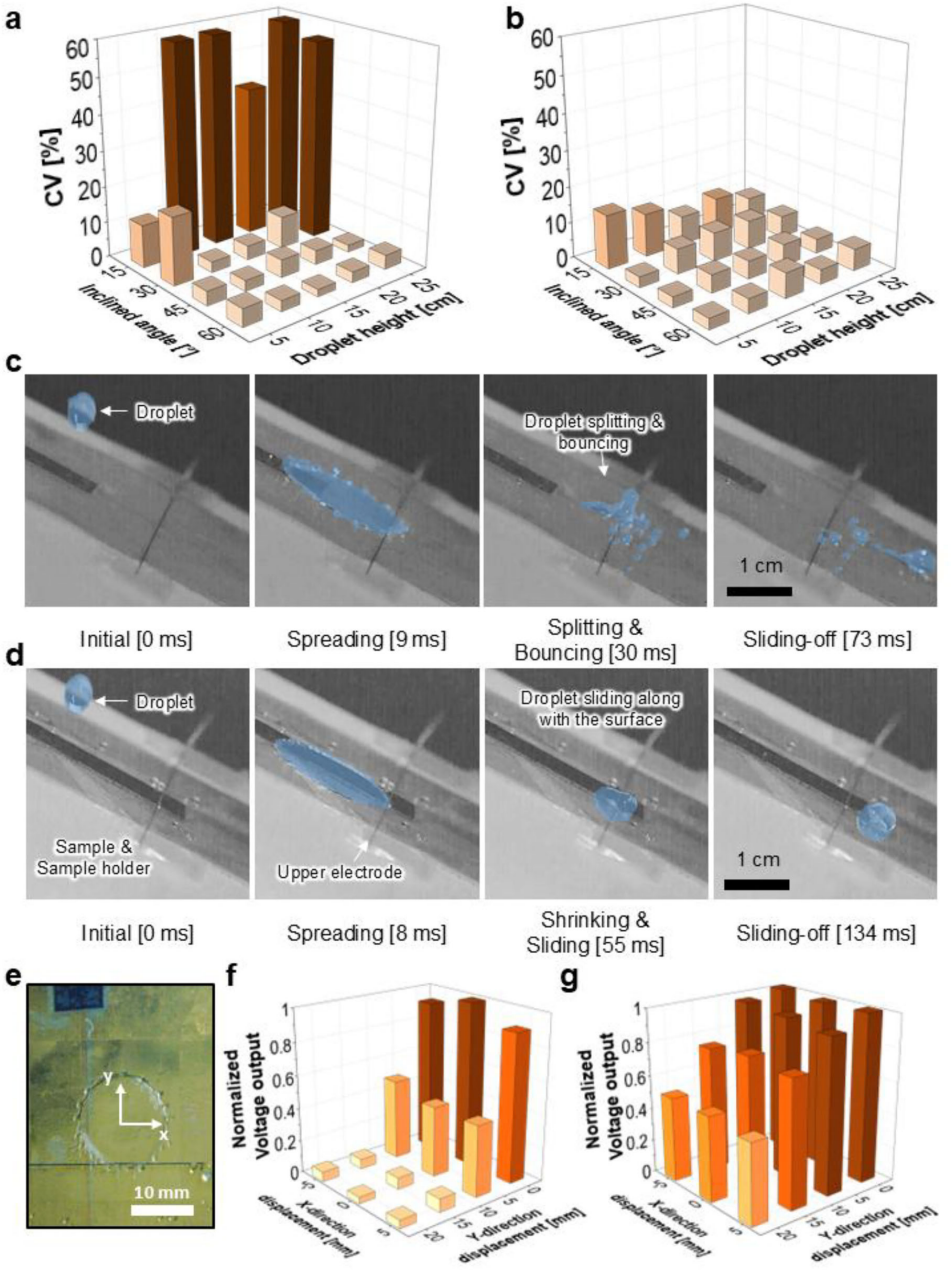
Measurement of the electrical voltage output stability of superhydrophobic and PDMS‐SCAL‐DEG platforms under various test conditions. Graphs show the uniformity of the generated output voltage according to the water droplet impact conditions (droplet release height (*h*) and inclination angle (*θ*) of the sample) of (a) superhydrophobic‐DEG and (b) PDMS‐SCAL‐DEG. *CV* represents the coefficient of variation of the open‐circuit voltage output. Visualization of water droplet behavior on (c) the superhydrophobic‐DEG and (d) the PDMS‐SCAL‐DEG surfaces using a high‐speed camera at *h* = 25 cm and *θ =* 30°. (e) Photograph image of the superhydrophobic‐DEG at the optimal point. Drop‐location‐sensitivity of (f) the superhydrophobic‐ and (g) PDMS‐SCAL‐DEG platforms. *h* and *θ* were 25 cm and 45°, respectively, in (e), (f), and (g). DI water is used with a volume of ∼ 49 µL in all droplet impact experiments. Uncertainties in (a) and (b) represent the standard deviation of the total 9 independent electrical measurements on the three samples. Uncertainties in (f) and (g) represent the standard deviation of the total 6 independent electrical measurements on the two samples.

On the other hand, the use of a PDMS‐SCAL‐DEG could be a suitable solution to address the challenges associated with superhydrophobic‐DEGs. Figure S5a,b shows scanning electron microscope (SEM) and atomic force microscope (AFM) images of the developed PDMS‐SCAL‐DEG, which clearly demonstrate its smooth surface characteristics. In particular, the PDMS‐SCAL surface has a very low roughness of 4.1 ± 1.0 nm, which is an essential requirement for suppressing the pinning of the three‐phase contact line and achieving surface sliding characteristics [28]. Additionally, the smooth surface characteristics can suppress the air compression phenomenon that occurs when a droplet impacts the surface, thereby minimizing the resulting repulsive force [31]. Figure S5c shows a cross‐sectional, tilted‐view SEM image of the PDMS‐SCAL‐DEG, confirming that the SiO_2_ intermediate layer and the PDMS‐SCAL layer are thinly deposited, with a thickness of approximately 116 ± 13 nm. We additionally performed AFM force‐curve analysis to estimate the thickness of the covalently attached PDMS‐SCAL brush layer. Specifically, we adopted an approach reported by Zhou et al. [37], using the jump‐in distance in the approach curve. In that method, the thickness is estimated from the displacement interval associated with the AFM tip interacting with the soft PDMS layer before reaching the underlying rigid surface. From 15 independent AFM force‐curve measurements, the PDMS‐SCAL brush thickness was estimated to be 4.25 ± 0.94 nm, as shown in Figure S6, which is consistent with the nanometric thickness range reported for PDMS‐SCAL coatings [30, 38]. Additionally, the PDMS‐SCAL‐DEG exhibits a static contact angle of 108.6 ± 3.9°, and advancing and receding CA were 108.8 ± 0.5° and 105.9 ± 0.7°, respectively. Such a low CAH (2.8 ± 0.8°) is due to the natural hydrophobicity of PDMS and its low surface roughness (Figure S5d) [39]. The chemical composition of PDMS‐SCAL‐DEG is shown in Figure S5e. Specifically, the atomic percentages of C, O, and Si elements are approximately 49.5%, 22.8%, and 27.8%, respectively. Although we used a recipe designed to produce PDMS brushes, it is crucial to provide direct evidence that the intended PDMS molecular structure was successfully formed on the surface. We additionally measured the Si 2p X‐ray photoelectron spectroscopy (XPS) narrow peak to provide direct chemical evidence that the PDMS‐SCAL‐DEG surface possesses PDMS‐like siloxane chemistry. As shown in Figure S7, in the absence of the PDMS‐SCAL coating, only the SiO_2_ peak (103.5 eV) was observed. On the other hand, for the SiO_2_‐PDMS‐SCAL coating, a peak of C_2_‐Si‐O_2_ (102 eV) was observed together with the SiO_2_ peak (103.5 eV), which means PDMS successfully formed on the surface during the PDMS‐SCAL coating process. We have also confirmed that these findings are consistent with results reported in other studies involving PDMS brushes that utilized similar recipes [40].

The hydrophobicity naturally facilitates droplet contact and detachment. In addition, the PDMS‐SCAL surface has slippery characteristics (sliding angle of 4.2 ± 0.7°) due to the minimized local slope change caused by the smooth surface roughness [28] and the freely movable covalently bonded PDMS brush [27, 29]. Due to these characteristics, the PDMS‐SCAL‐DEG has an excellent energy harvesting performance of 162.2 ± 3.8 V (at *h* = 25 cm and *θ* = 45°) as shown in Video S2, Figure S5f, g. Although the *E*_out_ of the PDMS‐SCAL‐DEG is approximately 0.97 µJ, which is slightly lower than that of the superhydrophobic‐DEG (1.30 µJ) with a PTFE‐based dielectric layer, it is confirmed to have excellent energy harvesting characteristics, as shown in Figure S5h. The difference in voltage output is primarily attributed to the distinct surface material of the PDMS‐SCAL and the superhydrophobic surface. The PDMS‐SCAL surface is made of PDMS brushes terminating with hydrocarbon groups (‐CH_3_). In contrast, the superhydrophobic surface is made of PTFE nanoparticles terminating with fluorinated groups (‐CF_3_). Because fluorine‐based materials possess high electronegativity and a high dielectric constant, they significantly enhance contact electrification [41, 42, 43].

The current energy output likely results from a combination of hydrodynamic and triboelectric properties of materials. Therefore, it needs to decouple these influences to better understand and optimize the PDMS‐SCAL‐DEG. To isolate the triboelectric properties, we designed a solid‐solid contact electrification experiment using a pushing tester (JIOCS‐120, Junil Tech) to maintain a constant 10 N force, 3 cm × 3 cm contact area, and 1.25 Hz frequency. Each DEG sample was mounted to the base with aluminum as the counter‐material. The results showed the PDMS‐SCAL‐DEG produced peak voltages of 75.87 ± 2.97 V, while the superhydrophobic‐DEG reached 256.86 ± 22.53 V, as shown in Figure S8. This indicates that the superhydrophobic surface material (PTFE nanoparticles with Teflon) has a higher electron affinity than the PDMS‐SCAL material (PDMS), which is advantageous for DEG energy generation. It can explain why the superhydrophobic‐DEG exhibits greater efficiency than the PDMS‐SCAL‐DEG. However, driven by the environmental concerns of fluorinated materials, recent research has shifted toward fluorine‐free DEGs. Our PDMS‐SCAL‐DEG aligns with this trend [44, 45, 46].

Although the open‐circuit voltage output of PDMS‐SCAL‐DEG is lower than that of superhydrophobic‐DEG, it still has a high energy harvesting performance, due to the PDMS (a coating material) having a large surface HOMO‐LUMO gap state (around 8 eV, similar to that of PTFE), which allows it to obtain electrons from water. The electrons alter the electronic structures and charge distributions, resulting in a net charge density difference in PDMS. As a result, PDMS can have a highly negatively charged surface, which is beneficial to DEG performance [47]. Moreover, the relatively high surface energy of PDMS‐SCAL‐DEG enables a larger droplet contact area (2.49 cm^2^) during droplet impact compared to a superhydrophobic‐DEG (2.15 cm^2^). It leads to a greater number of dipole or ion arrangements, and thus comparable energy harvesting performance is observed, even though the PDMS‐SCAL‐DEG is made of fluorine‐free materials [4]. It should be noted that the advantage of the PDMS‐SCAL coating over conventional crosslinked PDMS coatings does not arise simply from using PDMS as the coating material. The PDMS‐SCAL coating combines two key features: the inherent chemical properties of the PDMS material and a covalently bonded, liquid‐like brush structure. As we discussed above, the chemical nature of the PDMS material provides a triboelectrically active surface through enhanced liquid‐solid contact electrification while remaining fluorine‐free. Additionally, the covalently bonded, liquid‐like PDMS brush structure creates a slippery, nanoscale‐smooth interface that minimizes pinning. This slippery brush structure facilitates rapid droplet removal from the surface while suppressing droplet rebound, splitting, and the formation of residual droplets, thereby enhancing droplet output and operational stability. Additionally, the liquid‐like PDMS reduces frictional energy loss during droplet spreading, thereby altering droplet impact dynamics and the average rate of change in the impact area. Because the open‐circuit voltage output of a DEG depends heavily on the droplet contact area and its average rate of change [4, 20], the unique characteristics of liquid‐like PDMS are highly favorable for enhancing DEG energy output. Therefore, the PDMS‐SCAL‐DEG platform we developed enhances the stability of electrical output and reduces sensitivity to droplet landing positions by controlling the hydrodynamic behavior of droplets, while maintaining fluorine‐free triboelectric functionality. On the other hand, the underlying SiO_2_ has substantial charge storage and retention capabilities, as widely used in energy harvesting and electronic applications [48, 49, 50]. To verify this point, we additionally compared peak voltage output performance according to SiO_2_ intermediate layer thickness (10 nm, 100 nm, and 1 µm), as illustrated in Figure S9. However, the variations in performance resulting from the different thicknesses of the SiO_2_ intermediate layer were negligible. We believe this result is reasonable because the overall capacitance of a multilayer dielectric stack is governed by the series‐capacitance relation and is dominated by the much thicker PE and PET layers (∼149 µm and ∼51 µm), whereas the 10nm ‐ 1 µm SiO_2_ intermediate layer is too thin to meaningfully alter the equivalent dielectric thickness of the device.

Figure 2b represents the stable performance of energy generation caused by the conflicting characteristics of the PDMS‐SCAL‐DEG (relatively low water repellency but high droplet removal ability due to slippery characteristics). Like the superhydrophobic‐DEG, the plot represents the uniformity of energy generation across *θ* = 15°–60° and *h* = 5 – 25 cm. Although the open‐circuit voltage output may vary with *h* and *θ*, it was confirmed that the PDMS‐SCAL‐DEG exhibits stable performance under all conditions, with *CV* values of 15% or less. In other words, while the superhydrophobic‐DEG shows large output variation under high‐impact and low‐inclination conditions, where droplet rebound and splitting are more pronounced, the PDMS‐SCAL‐DEG maintains low *CV* values ​​over the same operating window because the smooth, low‐pinning PDMS‐SCAL surface suppresses droplet rebound and splitting while promoting coherent spreading and sliding. These stable characteristics are attributed to the fact, as explained above, that the PDMS‐SCAL‐DEG has a nanometrically smooth, covalently attached PDMS brush surface. This smooth PDMS‐SCAL surface does not provide texture‐confined air pockets like the superhydrophobic surface. Although a transient air film may still exist during droplet impact on smooth surfaces [51], the absence of roughness‐confined air pockets suppresses the gas‐spring‐like recoil pathway. Together with the low‐pinning nature of the PDMS‐SCAL interface, this allows the droplet to spread, retract, and slide coherently rather than splitting or bouncing.

A high‐speed camera video was used to visualize the droplet behavior at *h* = 25 cm and *θ* = 30° on the superhydrophobic‐ and PDMS‐SCAL‐DEG surfaces (Figure 2c,d). As described above, the falling droplet impacts the surface and goes through a spreading and retraction process. During impact, droplet splitting and bouncing can significantly limit its contact with the upper electrode. In addition, Figure S10 shows visualized images captured using a high‐speed camera with a lower droplet volume (15 µL droplet volume, 25 cm droplet initial release height, and a surface inclination angle of 45°). The impact due to the momentum of the traveling droplet is converted only into droplet rebound without droplet splitting, indicating that the droplet does not contact the upper electrode. In this case, the droplet rolls off without generating any electricity. On the other hand, it was confirmed that the droplet on the PDMS‐SCAL‐DEG followed the same steps as a superhydrophobic surface (from droplet impact to spreading and retraction). However, the droplet was observed to slide along the surface and contact the upper electrode without splitting and bouncing.

The results thus far indicate that the energy‐harvesting performance of DEGs depends on the initial droplet release height and location, and that an optimal point exists to maximize performance. The optimal point is determined by adjusting the droplet release location so that the droplet reaches its maximum spreading area while maintaining triple contact between the water droplet, the upper electrode, and the DEG surface. It should be noted that the optimal point (*y* = 0) was experimentally confirmed under each test condition. For example, while this point varied with experimental conditions, the optimal droplet impact location was ∼6.5 mm above the upper electrode in the y‐direction under a 49 µL droplet, a 25 cm drop height, and a 45° sample inclination angle on a PDMS‐SCAL‐DEG sample. The existence of the optimal droplet release point to maximize the energy harvesting performance has been frequently discussed in previous studies and has been identified as significantly reducing the applicability of superhydrophobic‐DEGs [18, 19]. Considering the initial droplet release location in nature is random (e.g., rainfall), the performance of superhydrophobic‐DEGs will be inefficient in real situations. Therefore, the challenges associated with performance degradation that place a high dependence on droplet release location need to be addressed to enable the ubiquitous application of DEGs. In this regard, we additionally generated the 3D graph images (Figure 2e–g) that compare the drop‐location sensitivity of both superhydrophobic‐ (Figure 2f) and PDMS‐SCAL‐DEG (Figure 2g) platforms. These illustrate the degradation in performance resulting from displacements in the x‐ and y‐directions, relative to the optimal point (Figure 2e), where the open‐circuit peak voltage output is maximized. Specifically, for both the superhydrophobic and PDMS‐SCAL‐DEG platforms, the difference in peak voltage output performance resulting from displacement along the x‐axis is negligible. Conversely, we observed a dramatic difference in performance caused by even slight displacements along the y‐axis (the direction away from the upper electrode). It should be noted that displacements in the x and y directions are defined relative to the sample itself. Since the sample is inclined at a 45° angle relative to the ground, the y‐axis displacement represents the actual displacement relative to the ground multiplied by the square root of 2.

In the case of the superhydrophobic‐DEG, a y‐axis displacement of merely 7.1 mm causes the voltage output performance to drop to less than half of its optimal value. Furthermore, at a displacement of 14.1 mm or greater, the energy harvesting performance declines by 92%–96% compared to the optimal point. In contrast, in the case of the PDMS‐SCAL‐DEG, performance degradation remains negligible compared to the optimal point when displaced by up to 7.1 mm along the y‐axis. As the y‐axis displacement increases to 14.1 mm and 21.2 mm, performance declines by 21%–25% and 49%–52%, respectively, demonstrating the excellent drop‐location insensitivity of the PDMS‐SCAL‐DEG. Even though the PDMS‐SCAL‐DEG shows reduced energy harvesting performance compared with that of superhydrophobic‐DEG, the droplet location insensitivity is preferred for overall performance. It will minimize the energy loss when the drop location is far from the optimum point, resulting in robust DEG operation. Furthermore, we investigated the effect of y‐directional displacement at the drop location (relative to the optimal point (*y* = 0)) on the short‐circuit current and transferred charge, as shown in Figure S11. The results revealed that for the superhydrophobic‐DEG, even a small displacement of 7.1 mm led to a significant reduction in current and corresponding charge output, by 42% and 49%, respectively, compared to the optimal point. Moreover, as the displacement increased beyond 14.1 mm, both values reduced to less than 7% of their optimal levels (Figure S11a). In contrast, the PDMS‐SCAL‐DEG maintained performance levels exceeding 94% for current and 98% for charge at a y‐directional displacement of 7.1 mm and continued to sustain performance above 50% even at 14.1 mm (Figure S11b).

We further performed quantitative high‐speed camera analysis focusing on droplet‐location sensitivity (at y‐direction displacement of 0, 7.1, and 14.1 mm from the optimal location) as shown in Figure S12, Videos S3 and S4. We first investigated the contact area over time for both PDMS‐SCAL‐DEG and superhydrophobic DEG, starting at the moment of contact with the upper electrode. For the PDMS‐SCAL‐DEG, the droplet contact area reaches its maximum at the optimum point (*y* = 0) [18]. As the y‐direction displacement increases, the contact area decreases at any given timestamp. Upon initial contact with the upper electrode, the areas were 2.49, 2.40, and 2.02 cm^2^ for *y* = 0, 7.1, and 14.1 mm, respectively. Since the DEG open‐circuit voltage output is governed by both the droplet contact area and its average rate of change [19, 20], we analyzed these dynamics over a 25 ms interval from 0 ms. The average rates were 0.079, 0.078, and 0.062 cm^2^/ms for *y* = 0, 7.1, and 14.1 mm, respectively, as shown in Figure S12a. The minimum change in maximum contact area and average rates between *y* = 0 mm and 7.1 mm suggests that a stable voltage output can be obtained even with the offset. Furthermore, the more significant reduction in both parameters at *y* = 14.1 mm supports the observed degradation in voltage output (Figure 2g). In contrast, for the superhydrophobic DEG, it is challenging to measure temporal variations in the droplet contact area due to satellite droplet formation and rebounding during impact. Consequently, only the maximum contact area at *y* = 0 mm could be measured (2.15 cm^2^). It should be noted that the value presented above measured the spreading area observed from the top view, and the actual contact area with the surface might be smaller due to droplet bounce. Unlike the PDMS‐SCAL‐DEG, the rebounding behavior and satellite droplet formation hinder effective contact with the upper electrode in superhydrophobic DEG. To quantify these effects, we used side‐view imaging, as shown in Figure S12b, to analyze rebound behavior. The rebound heights were 1.67, 2.5, and 3.75 mm for *y* = 0, 7.1, and 14.1 mm, respectively. Notably, at y = 0 mm, rebounding occurs only at the droplet edges, whereas the entire droplet rebounds at *y* = 7.1 and 14.1 mm. Top‐view imaging was also employed to count satellite droplets, yielding 7, 7, and 14 droplets for *y* = 0, 7.1, and 14.1 mm, respectively (Figure S12c). The corresponding droplet dynamics are provided in Videos S5 and S6. These findings confirm that rebounding behavior and increased satellite droplet formation drive the voltage output degradation as the y‐direction displacement increases.

### 2.2 Electrical Characteristics of PDMS‐SCAL‐DEG

The energy harvesting performance of PDMS‐SCAL‐DEG is further characterized under various conditions. The effect of external resistance is analyzed by connecting resistors in parallel with the PDMS‐SCAL‐DEG (Figure 3a). The corresponding peak power is calculated based on the peak voltage measurement. The detailed calculation is explained in the Materials and Methods section. The peak power density was calculated by identifying the maximum peak power across a range of load resistors from 1 kΩ to 56 MΩ (equivalent to 1 kΩ to 43.8 MΩ when accounting for the passive probe's input resistance). As shown in Figure 3a, the maximum peak power reached 28.46 mW at 50 kΩ, which indicates that the electrical impedance of the PDMS‐SCAL‐DEG is 50 kΩ. The resulting power density depends on the maximum droplet contact area, without the upper electrode (2.49 cm^2^), which was adopted to normalize the power density, consistent with previous reports [4, 8, 52] (Figure 3a). Furthermore, we have also calculated the power density using the maximum electrode overlap area (2.59 cm^2^), defined as the sum of the upper electrode area and the maximum droplet contact area, and the device footprint (35 cm^2^). Consequently, the maximum peak power densities are calculated as 116.4, 111.9, and 8.3 W/m^2^ when normalized by the maximum droplet contact area, electrode overlap area, and device footprint, respectively (at 50 kΩ). Among these metrics, the maximum droplet contact area is widely adopted in previous DEG studies because it directly reflects droplet impact dynamics and the energy‐harvesting efficiency per droplet. On the other hand, normalization based on the device footprint is highly meaningful for practical applications. This metric represents the actual physical space required to capture ambient water resources such as rainfall and dew. In this study, we presented the various metrics to provide a comprehensive evaluation of DEG performance.

**FIGURE 3:**
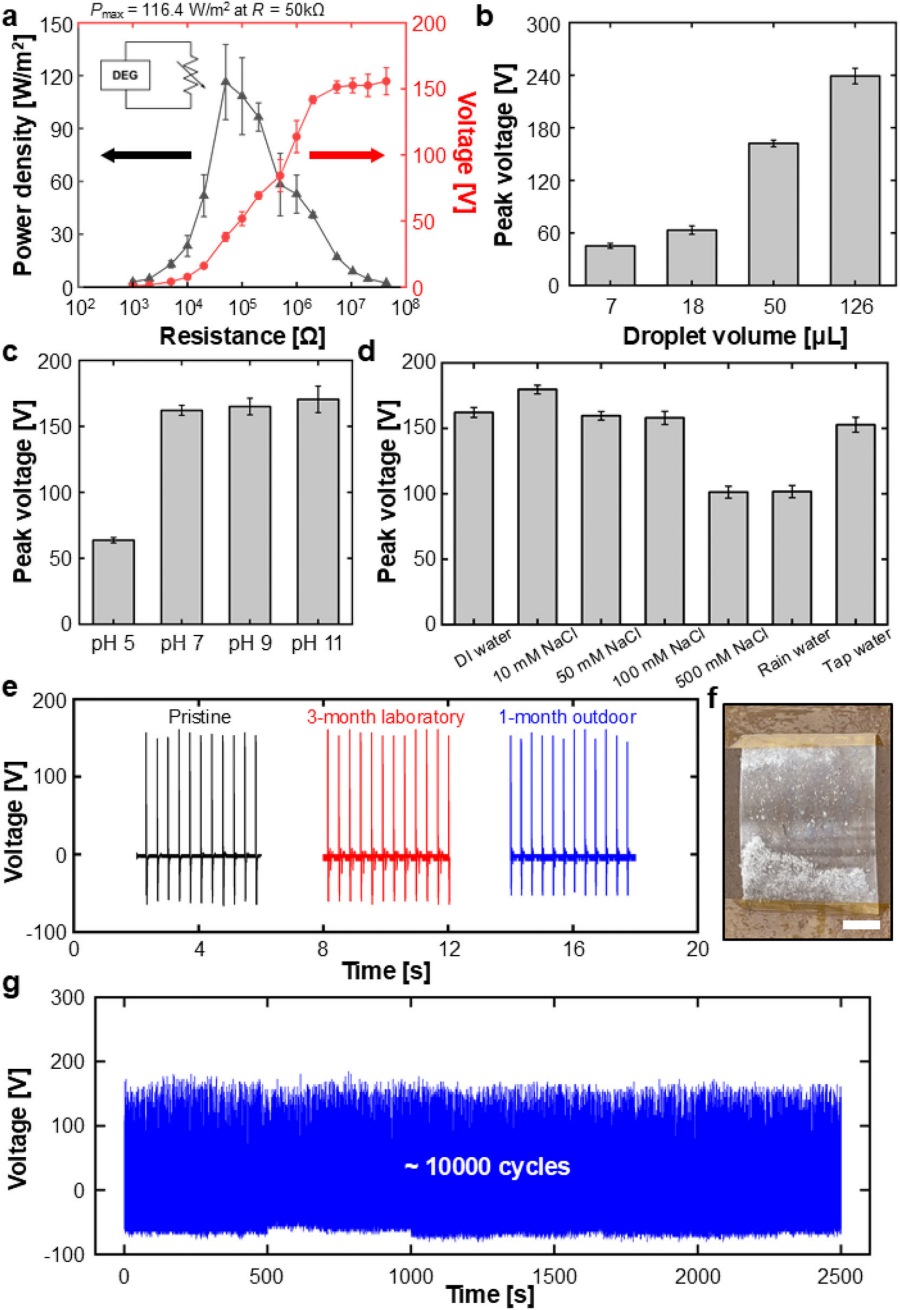
Characterization and durability of the PDMS‐SCAL‐DEG's energy harvesting performance. (a) The average peak power density and voltage according to the load (1 k‐43.8 MΩ). The load resistance values are adjusted to account for the input resistance of the passive probe. The peak power density was normalized by the active droplet contact area (2.49 cm^2^). Open‐circuit output peak voltage of PDMS‐SCAL‐DEG with different (b) droplet volume, (c) droplet pH, and (d) electrolyte. (e) Long‐term performance stability of the PDMS‐SCAL‐DEG surface. Open‐circuit voltage output performance results for the sample stored for 3 months in a laboratory environment (Red) and 1 month in an outdoor environment (Blue). (f) Photograph of an outdoor exposure test of the PDMS‐SCAL‐DEG sample. The PDMS‐SCAL‐DEG samples were attached to the exterior wall of the building for 1 month (Nov. 05, 2025 ∼ Dec. 05, 2025, Champaign, Illinois, US). The scale bar is 1 cm. (g) Energy harvesting performance stability test of the PDMS‐SCAL‐DEG surface. The open‐circuit output voltage remained stable over ∼10 000 cycles. Droplet height was 25 cm, and the inclined angle was 45°. DI water was used at a volume of ∼49 µL in all droplet impact experiments.

Interestingly, while analyzing the open‐circuit output voltage and the resulting harvested energy (Figures S2g,h and S5g,h), we observed that our developed DEGs (both superhydrophobic and PDMS‐SCAL) exhibit much larger energy contribution from the negative‐voltage stage than is typically emphasized in the previous literature, where the principal charge transfer and peak output are generally associated with droplet spreading to the maximum area and contact with the top electrode, whereas the subsequent retraction stage is mainly described as a charge backflow process [8, 18, 20]. DEG operation is characterized by two distinct time parameters: hydrodynamic time (*τ*_h_) and electrical relaxation time (*τ*_e_). *τ*_h_ represents hydrodynamics (droplet impact, spreading, and shrinkage) and is governed by the droplet's *Weber number (We)*, surface wettability, and inclination angle. While *τ*_e_ is determined by the circuit *R* and *C*. This implies that even under identical hydrodynamic conditions, the voltage output waveform can be significantly influenced by external resistance [19]. We compared the voltage output of PDMS‐SCAL‐DEG across various external resistances (5.6 MΩ, 11.2 MΩ, 22.4 MΩ, and 56 MΩ) and calculated the corresponding energy of each phase (Steps 2 and 3 in Figure S5f,g), as shown in Figure S13. The results indicate that the energy portion of Step 3 increases from 15.4% to 28% as the external resistance rises. Given that Step 3 lasts considerably longer than Step 2 (*τ*_h_ of 64.4 ms vs 7.9 ms, respectively, under fixed conditions of 25 cm release height, 45° inclination angle, and 49 µL volume), a higher external resistance (increased *τ*_e_) more effectively captures the induced charge movement occurring over the hydrodynamic timeframe. Furthermore, the voltage outputs were measured using a passive probe with an input resistance of 200 MΩ. Therefore, the energy portion of Step 3 reached 38.5%.

Furthermore, the effect of droplet volume on peak voltage is analyzed, demonstrating an increasing trend with higher droplet volume (Figure 3b). In droplet dynamics, the spreading area during droplet impact increases with increasing *Weber number (We)* [13]. As the droplet volume increases, the *We* also increases, leading to a wider spreading area and promoting the movement of induced charges during PDMS‐SCAL‐DEG operation. Therefore, higher energy harvesting performance is observed with larger droplet volumes. Thanks to their slippery surface properties, very small impacting droplets can also be easily removed from PDMS‐SCAL‐DEG surfaces, allowing them to operate with droplet volumes lower than 10 𝜇L (Figure 3b). Although a considerably high peak voltage output at small droplet volumes is also observed in superhydrophobic‐DEG (Figure S14), its practical operating stability is limited when droplet impact conditions deviate from the optimum. Considering the potential applications of the PDMS‐SCAL‐DEG, its wide range of operating droplet volumes indicates that it is an appropriate water‐energy harvester for real‐world applications.

To further demonstrate versatility, the effect of the droplet constituent also needs to be investigated. As shown in Figure 3c, the peak voltage increases with increasing droplet pH, as reported previously [53]. Furthermore, Figure 3d shows the effect of the electrolyte concentration. When a small amount of NaCl is added (10 mM), the peak voltage increases to 179.7 ± 3.3 V (DI water: 162.2 ± 3.8 V). However, when the molar concentration of NaCl increases (500 mM), the energy harvesting performance decreases significantly (101.3 ± 4.6 V). This implies that slightly dissolved Na^+^ and Cl^−^ ions can participate in the operation mechanism of the PDMS‐SCAL‐DEG, but excessive ion concentration can interrupt contact electrification [11]. These results indicate the possibility of further enhancing energy harvesting performance through the competition relationship, and finding the optimal electrolyte concentration could be another strategy to improve the PDMS‐SCAL‐DEG energy harvesting performance. The peak voltages of rain water (101.7 ± 4.7 V) and tap water (152.8 ± 5.7 V) are also measured to assess their energy‐harvesting potential. Even though their peak voltages are lower than those of DI water, the results indicate that PDMS‐SCAL‐DEG is qualified to harvest the potential energy of water more relevant to real life. Additionally, to confirm the excellent durability of the PDMS‐SCAL‐DEG platform, we compared the output voltage performance after 3 months of storage in a laboratory environment and after 1 month of outdoor exposure. The red and blue peaks in Figure 3e represent the output voltage performance after 3‐month laboratory storage and 1‐month outdoor exposure, respectively, showing no performance degradation compared to the pristine one (black). Notably, during the 1‐month outdoor exposure test, 10 precipitation events (including snow and rain) occurred, and temperatures ranged from 24°C to −17°C. Figure 3f showed snow accumulating on the samples, indicating that they were exposed to harsh conditions. Notably, thanks to PDMS‐SCAL‐DEG's excellent anti‐fouling characteristics, no water stains remained on the sample surface after the melted snow had completely evaporated. Despite wide variations in temperature and humidity and long‐term exposure to UV, the PDMS‐SCAL‐DEG maintained its energy‐harvesting performance stably without degradation. It should be noted that the PDMS‐SCAL‐DEG samples were tested for energy‐harvesting performance immediately after outdoor exposure, without any cleaning. This indicates that the anti‐fouling properties conferred by the slippery characteristics of the PDMS‐SCAL‐DEG minimized performance degradation caused by surface contamination. Finally, the long‐term operating stability of PDMS‐SCAL‐DEG's energy‐harvesting performance is investigated. Over ∼10000 cycles, PDMS‐SCAL‐DEG maintains its performance well, as shown in Figure 3g. These results clearly demonstrate the long‐term durability of the PDMS‐SCAL‐DEG surface.

### 2.3 Electrical Device Application and Field Validation of PDMS‐SCAL‐DEG

To demonstrate the application of the developed PDMS‐SCAL‐DEG, experiments were conducted to enable LED lighting and capacitor charging. Figure 4a shows that the PDMS‐SCAL‐DEG, when operated by a single droplet, delivers sufficient energy to power 100 LEDs simultaneously. Figure 4b shows that a 4.7 𝜇F capacitor is charged by the PDMS‐SCAL‐DEG operation within 100 s. To charge the capacitor, the PDMS‐SCAL‐DEG device was connected to a rectifier that converts the alternating current (AC) output to direct current (DC) energy. The charging performance of the PDMS‐SCAL‐DEG is also evaluated at *y* = 14.1 mm. Note that during 100 s of charging, the charging voltage provided by the PDMS‐SCAL‐DEG decreases by only about 24% compared to when it operates at the optimum droplet release point. For the superhydrophobic DEG, we observe a reduction of approximately 91% (Figure S15). This finding supports the notion that the PDMS‐SCAL surface effectively reduces the sensitivity of DEG performance to the initial droplet release location. Nevertheless, the low energy harvesting output of a single DEG cell still limits its application to various devices. Therefore, we aimed to effectively increase the energy harvesting output by connecting DEG cell arrays in parallel, enabling the application to a wider range of electrical devices. Figure 4c shows the electrical circuit diagram for charging capacitors in parallel through independent DEG cells. It demonstrates that our developed PDMS‐SCAL‐DEG platform, like other DEGs, is capable of multi‐array use, allowing for expansion to devices requiring higher operating voltages (Figure 4d), including a thermostat (Digital LCD Thermometer, HiLetgo) and a calculator (SL‐300SV, Casio). Figure 4e shows the capacitor charging characteristics in a multi‐DEG array. Using 4 DEG cells, we measured the charging capability up to 3.47 V in 100 s. The superior release location‐insensitivity and the multi‐DEG array applicability of PDMS‐SCAL‐DEG indicated that the device can be an attractive selection for an excellent, versatile energy supply device for operating small electronic devices. We further investigated the environmental compatibility of the PDMS‐SCAL‐DEG platform. As shown in Figure 4f, the photograph taken outside with the PDMS‐SCAL‐DEG in front of the camera sensor shows visible transparency. The visible light transmittance, as quantified using a UV–vis‐NIR spectrophotometer, shows that the PDMS‐SCAL‐DEG exceeds 80% transmittance in the 470 to 900 nm wavelength range (Figure 4g). Figure 4h indicates the flexibility of the PDMS‐SCAL‐DEG. The results suggest that the PDMS‐SCAL‐DEG could be a candidate for a ubiquitous energy harvester where droplet contact and separation occur, such as on windows, rooftops, and the tops of photovoltaic panels.

**FIGURE 4:**
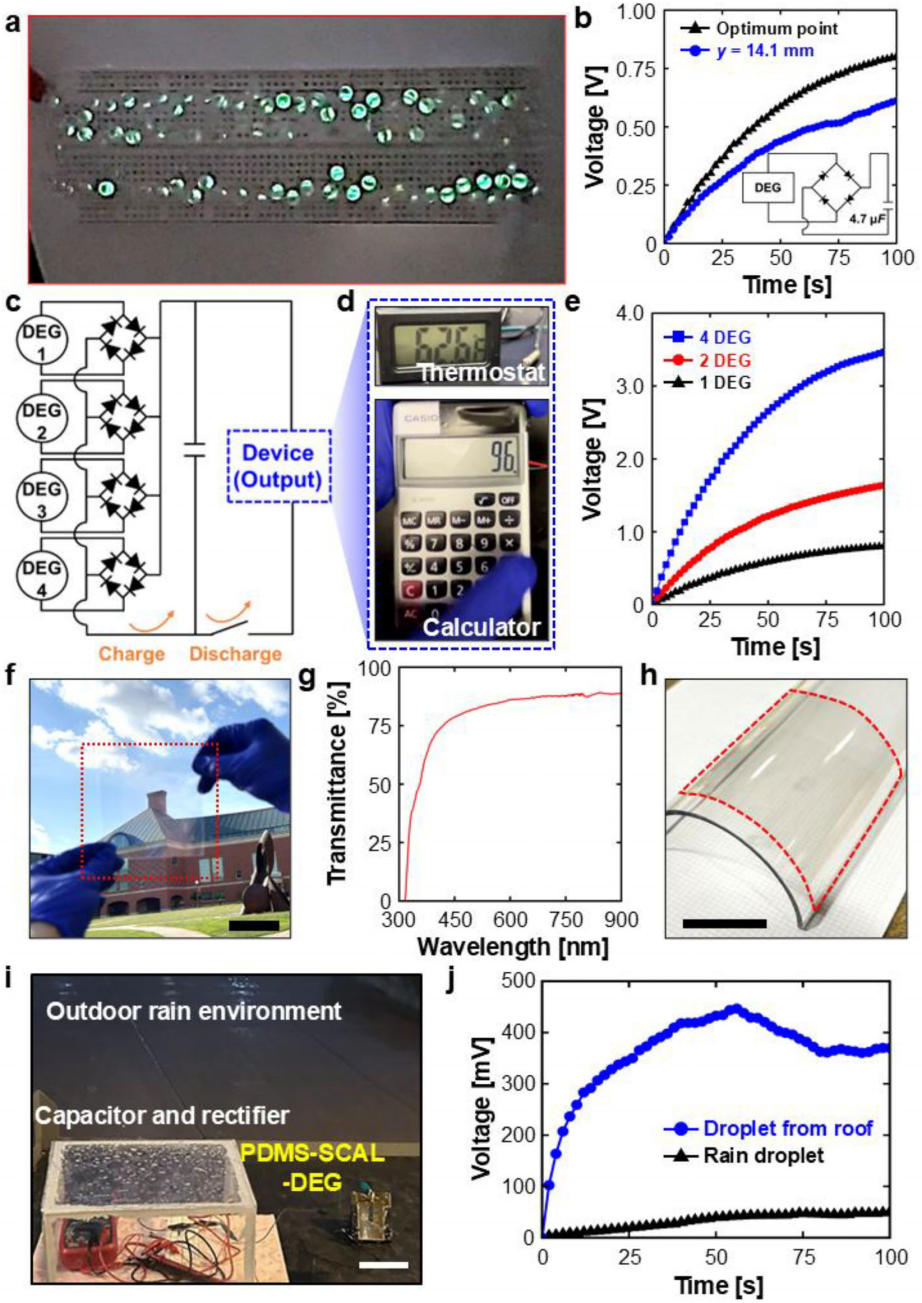
Applications of the PDMS‐SCAL‐DEG platform. (a) Photograph demonstrating the lighting of 100 commercial LED lights by a PDMS‐SCAL‐DEG. (b) Capacitor charging performance of a PDMS‐SCAL‐DEG with *h* = 25 cm and *θ* = 45°. Here, *y* is the y‐directional displacement from the point where the DEG performance is maximum. The inset is an electric circuit diagram of a capacitor charging. (c) Electrical circuit diagram of the multi‐DEG array of PDMS‐SCAL‐DEG. (d) Photographs of small electronic devices (a thermostat and a calculator) can be powered by a multi‐DEG array of PDMS‐SCAL‐DEG. (e) Capacitor charging performance of the DEG array according to the number of operating PDMS‐SCAL‐DEGs. Droplet height was 25 cm, and the inclined angle was 45°. (f) Photograph of the PDMS‐SCAL‐DEG showing high visible transparency and its (g) measured transmittance as a function of light wavelength. The scale bar is 5 cm, and the photograph is of the Grainger Library on the University of Illinois Campus. (h) Photographs of the PDMS‐SCAL‐DEG attached to a curved surface, showing mechanical flexibility. The scale bar is 5 cm. (i) Photograph of the test setup for the DEG performance evaluation, and (j) capacitor charging voltage of PDMS‐SCAL‐DEG with *θ* = 45° in the field validation test under an actual rainfall environment. The results of ‘droplet from roof’ and ‘rain droplet’ were obtained at 2 AM, Mar./05/2026 (∼6.3 mm precipitation) and 8 PM, Mar./15/2026 (∼9.4 mm precipitation), respectively, at the outdoor of Grainger Library on the University of Illinois Campus. The scale bar is 10 cm.

In previous studies, practical rainfall energy harvesting has been demonstrated in DEG devices and related droplet‐based liquid‐solid electrification platforms using simulated rainfall environments [54, 55]. However, to quantitatively reproduce natural rainfall using a drop‐ or spray‐type simulator, it is not sufficient to provide continuous water input alone, but it should also reproduce the drop‐size distribution, terminal velocity, rainfall intensity, spatial uniformity, and kinetic energy [56]. Prior reports emphasized that these parameters must be considered together, and that many laboratory simulations still deviate from natural rainfall, especially in drop‐size distribution and kinetic energy [57]. For this reason, rather than adding an insufficiently calibrated benchtop spray test that could introduce an artificial rainfall condition, we chose to perform field validation under actual rain events. Accordingly, we performed the capacitor‐charging tests for the PDMS‐SCAL‐DEG connected to the same rectifier‐capacitor circuit (Figure 4i) under two conditions: (i) direct exposure to actual rainfall, and (ii) “roof‐drip” conditions, where water naturally coalesced and dripped from a roof edge. Because actual raindrops fall randomly across the device and the DEG platforms are vulnerable to shorts by the water bridge near the sample boundary, we completely sealed the area surrounding the sample with silicone epoxy. Additionally, we enclosed the measurement components, including the rectifier and capacitor, with a transparent acrylic container. Under direct rainfall conditions (during a rain event for which nearby Willard Airport observations recorded ∼9.4 mm of precipitation), the capacitor voltage reached 51.9 ± 15.0 mV after 100 s (Figure 4j), which corresponds to only 6.1% of the laboratory charging voltage obtained with the same circuit and measurement duration (849.7 ± 53.8 mV). We interpret this lower charging voltage as being consistent with natural rainfall being dominated by smaller and spatially scattered droplets than our laboratory benchmark condition [58]. In our study, the 49 µL laboratory droplet corresponds to a spherical‐equivalent diameter of ∼4.5 mm, whereas natural rainfall has been reported to span 0.2 – 5.1 mm, with most droplets lying in the 1 – 2 mm range [59]. In addition, the small, scattered raindrops make it difficult to simultaneously contact both the dielectric surface and the upper electrode, thereby generating unstable DEG output [54]. To further evaluate an application‐relevant condition with naturally coalesced water input, we also tested a roof‐drip configuration during another rain event (∼6.3 mm of precipitation in Willard airport weather observation history). In that case, the capacitor voltage reached 352.5 ± 21.0 mV after 100 s, that is, 6.8 times higher than under direct rainfall (Figure 4j). We believe that the roof‐drip condition is more relevant to the practical deployment of DEG. It should be noted that these field tests provide the practical validation of real‐weather input, although they don't replace a strictly measured and performed rain simulator study.

### 2.4 Comprehensive Durability Test and Future Perspective

To ensure sufficient durability for practical applications, we comprehensively evaluated PDMS‐SCAL‐DEG's durability through mechanical, chemical, and electrical robustness tests. Although the PDMS‐SCAL coating mainly consists of flexible PDMS brushes, we used a SiO_2_ intermediate layer to achieve robust covalent bonding, which is inherently brittle and not suitable for flexible devices [60]. Therefore, we verified the bending durability of PDMS‐SCAL‐DEG through a bending cycle test. Figure 5a,b present the bending test results and corresponding test images of the PDMS‐SCAL‐DEG platform, respectively. Our experiments confirmed that the performance of the DEG has no significant degradation throughout 1000 bending cycles at a bending radius of 7 mm. It should be noted that ITO films are also inherently brittle. We analyzed the change in electrical resistance of the ITO film as a function of bending radius, as shown in Figure S16a, to determine the bending radius for our bending‐cycle experiments. The results indicated that changes in the ITO film's electrical resistance performance were negligible when the bending radius was 6 mm or greater. Thus, we conducted the bending cycle tests using a bending radius of 7 mm. During the cyclic bending test, we protected the ITO film with Kapton tape, thereby minimizing any degradation in its performance. Figure S16b shows the SEM images of the ITO and PDMS‐SCAL films following a single bend at a bending radius of 5 mm. While micro‐cracks formed on the surface of the ITO film after just one bend at 5 mm, the PDMS‐SCAL (SiO_2_‐PDMS‐SCAL) film did not exhibit any micro‐cracks. This is attributed to the fact that the SiO_2_ layer (∼100 nm) is significantly thinner than the ITO film (∼2 µm), resulting in superior bending resistance [61]. Based on cyclic bending tests, we anticipate that the performance of PDMS‐SCAL‐DEG will remain stable up to the allowable bending radius (and cycle number) that the underlying ITO film can withstand.

**FIGURE 5:**
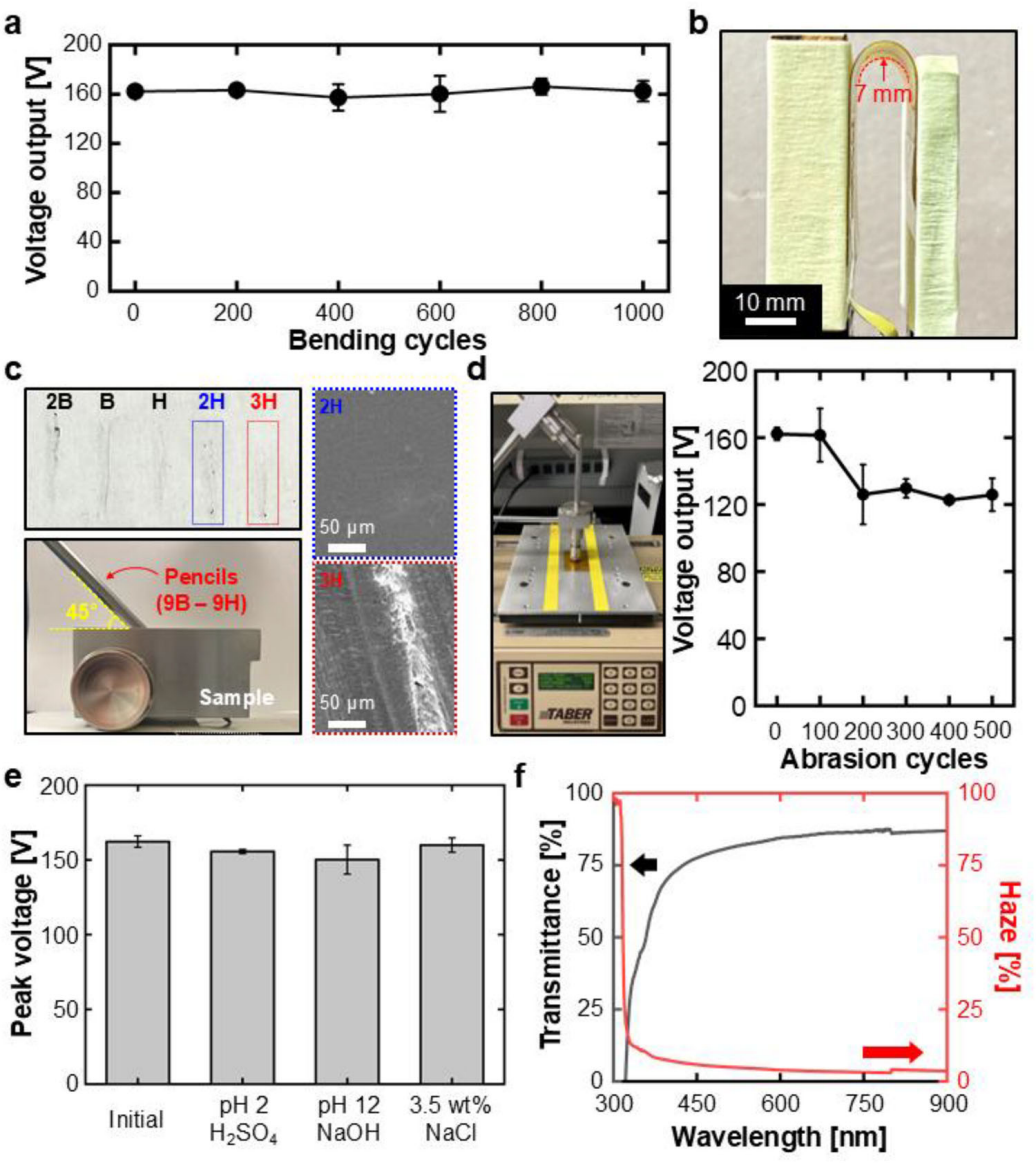
Comprehensive durability test results of PDMS‐SCAL‐DEG platform. (a) Bending cycle test results and (b) photograph image of the PDMS‐SCAL‐DEG at a bending radius of 7 mm. (c) Test results of the PDMS‐SCAL‐DEG surface after pencil hardness tests. (d) Photograph and test results of the surface abrasion test. (e) Open‐circuit output peak voltage of the PDMS‐SCAL‐DEG after immersing in the different chemical solutions (pH 2 H_2_SO_4_, pH 12 NaOH, and 3.5 wt% NaCl aqueous solutions) for 24 h. (f) Transmittance and haze measurement results after 1 month of outdoor exposure. During the DEG experiments, the droplets were dropped from *h* = 25 cm and *θ* = 45°. DI water is used with a volume of ∼49 µL in all droplet impact experiments. Error bars in (a) and (d) represent the standard deviation of 3 independent electrical measurements on the same sample. Error bars in (e) represent the standard deviation of the total 6 independent electrical measurements on the two samples.

Second, we evaluated the coating hardness using a pencil hardness test according to ASTM D3363. Figure 5c illustrates our experimental setup and results. It should be noted that, due to the excellent anti‐adhesion and slippery properties of the PDMS‐SCAL coating, it is visually difficult to assess the surface's pencil hardness. Therefore, we conducted a comparative analysis of SEM images, which revealed that PDMS‐SCAL‐DEG has a hardness of approximately 2H. We further conducted the abrasion tests using an automatic cyclic abrasion tester, as shown in Figure 5d. It should be noted that each cycle involved a 20 mm horizontal displacement, and given the extremely small abrasion stroke of 20 mm, the sample was rigidly fixed to ensure that the abrasion point remained consistent across every cycle. Furthermore, during the DEG performance evaluation, water droplets were dispensed precisely onto the designated abrasion point. The peak voltage output was measured every 100 cycles to monitor any degradation in performance resulting from abrasion. The results indicated that while the DEG performance remained well maintained up to 100 cycles, it declined by approximately 22% after 200 cycles. Additional abrasion testing further confirmed that, even after 500 abrasion cycles, the energy harvesting performance remained robust, retaining 75.6%–80.0% of its initial value. Abrasion damage on PDMS liquid‐like surfaces can occur via two possible pathways [62]: degrafting or scission of tethered PDMS chains and damage to the underlying layer, which can drastically reduce liquid‐repellent performance. Based on this perspective, the partial voltage decrease observed after the abrasion test may stem from localized damage to the covalently bonded PDMS brush layer or the SiO_2_ adhesion layer.

On the other hand, to verify the excellent chemical durability of the PDMS‐SCAL‐DEG platform, we immersed PDMS‐SCAL‐DEG samples for 24 h in H_2_SO_4_ (pH 2), NaOH (pH 12), and 3.5 wt% NaCl (simulating seawater) aqueous solutions, respectively, and subsequently measured the open‐circuit output peak voltage. The measurement results revealed only minimal performance degradation across all cases (4.1%, 7.4%, and 1.4% for H_2_SO_4_, NaOH, and 3.5 wt% NaCl aqueous solutions, respectively), as shown in Figure 5e. These experimental results confirm that PDMS‐SCAL‐DEG possesses excellent chemical stability for practical applications. For integration into various industrial devices, such as windows or solar panels, durability against ultraviolet (UV) radiation and dust is essential. To verify the optical durability, we additionally confirmed the optical performance for the 1‐month outdoor‐exposed PDMS‐SCAL‐DEG samples. Our measurement shows no significant difference in transmittance, which means the optical aging was minimal during the outdoor exposure, as shown in Figure 5f. In addition to the transmittance, we characterized the haze of PDMS‐SCAL‐DEG. Haze, defined as the ratio of transmitted light deviated from the incident light by more than 2.5°, quantifies light scattering. We measured haze at wavelengths from 300 to 900 nm using the same equipment used for transmittance. The result shows that the PDMS‐SCAL‐DEG maintained a haze below 7.3% over the 400 to 900 nm wavelength range (Figure 5f). We additionally characterized the surface morphology and wettability of the PDMS‐SCAL‐DEG after 1‐month outdoor exposure (Figure S17). The surface morphology, thickness, and roughness showed no obvious change after outdoor exposure, indicating that the PDMS‐SCAL coating and underlying device structure were largely preserved. The static water contact angle (107.7 ± 0.6°) was also maintained at a level comparable to that of the pristine surface. In contrast, the advancing and receding contact angles and sliding angle were 109.2 ± 0.4°, 101.8 ± 0.8°, and 15.6 ± 3.2°, respectively. Based on our measurements, the slight increase in CAH (7.4°) is mainly due to the reduction in the receding angle rather than a significant change in the advancing angle. We attribute this behavior to a moderate increase in contact‐line pinning, likely caused by local micro‐defects, trace contamination, or chemical non‐uniformity introduced during outdoor exposure. This interpretation is consistent with previous studies showing that CAH is highly sensitive to local surface roughness, chemical heterogeneity, adsorption/desorption, and contact‐line pinning [63]. In addition, UV/oxidative exposure of PDMS‐based surfaces can introduce hydrophilic functional groups or local chemical changes, which may further contribute to the reduction in receding angle [64].

We additionally provide a comprehensive comparison of previous works in Table 1, evaluating energy output, wettability, multi‐functionality (stretchability/flexibility and transmittance), and durability. We can directly compare the material aspects (fluorinated, liquid‐infused, and non‐fluorinated), electrical performance, multi‐functionality (transparency, flexibility), and durability. From a materials perspective, we compared existing literature on droplet‐based TENGs and DEGs across three categories: (i) studies with high peak output based on fluorinated surfaces, (ii) studies utilizing lubricant‐infused slippery interfaces to ensure the performance stability in the extreme conditions, and (iii) studies aiming to enhance sustainability through the use of non‐fluorinated or bio‐based materials. However, these prior studies typically meet only a subset of key criteria, such as output performance, eco‐friendliness, durability, and real‐world applicability. In contrast, the developed PDMS‐SCAL‐DEG in this study distinguishes itself by employing a fluorine‐free, covalently attached slippery interface that delivers relatively high output, insensitivity to droplet location, operational capability under actual rainfall conditions, and comprehensive durability. This work can be positioned as one of the most well‐balanced DEG designs to deploy, specifically engineered with practical deployment in mind.

**TABLE 1:** Summary of droplet‐based TENGs and DEGs’ characteristics. *I*_peak_, *V*_peak_, and *P*_peak_ represent peak current, peak voltage, and peak power (density), respectively. *P*_peak_ is calculated when the external load matches the impedance of each energy harvester. Its density is calculated based on the maximum droplet spreading area. CA, CAH, and SA represent the contact angle, contact angle hysteresis, and sliding angle, respectively. FEP represents fluorinated ethylene propylene, and PTFE represents polytetrafluoroethylene.

Author	Substrate	Energy output (*I*_peak_ / *V*_peak_ / *P*_peak_)	Wettability (CA/CAH/SA)	Multi‐functionality (Stretchability or flexibility, Transmittance)	Description including durability
Wu et al. [53]	Fluorinated DEG (FEP)	∼ 6 mA /—/ 160 W/m^2^	—	—	– Proposed a charge‐trapping‐based electricity generator (CTEG), utilizing a homogeneous electrowetting‐assisted charge injection method to pre‐charge a fluoropolymer film. – Demonstrated no degradation in performance during a 100‐day period of intermittent operation testing.
Wang et al. [9]	Fluorinated DEG (FEP)	0.4 mA / 200 V /—	168.5°	—	– Utilize a superhydrophobic surface to harvest energy from high‐frequency impinging droplets (165 Hz). – Demonstrated stable performance after 7 × 10^4^ droplet impact and 150 days of operation. Remained stable under 100% RH for a 30 min exposure.
Yoo et al. [4]	Fluorinated DEG (FEP)	1.5 mA / 184 V / 1.9 kW/m^2^	159° /—/ 7°	—	– Reported a lotus leaf‐inspired droplet‐based electricity generator (LL‐DEG) to achieve low‐adhesive superhydrophobicity – Demonstrated energy harvesting using small droplets with volumes as low as 6 𝜇L.
Zheng et al. [65]	Fluorinated DEG (PTFE)	1.25 mA / 150 V /—	—	−80% in the wavelength range of 400−900 nm	– Multilayered structures are integrated with organic photovoltaics to achieve all‐weather energy harvesting. – Record 25%‐ energy conversion efficiency with an external load of 1 GΩ.
Zhang et al. [66]	Fluorinated DEG (PTFE)	6.16 mA / 344 V / 2.03 kW/m^2^	—	—	– Designed a DEG through a comprehensive analysis of parameters, including dielectric layer thickness, droplet ion concentration, and external load. – Achieved high performance and rapid charging, requiring only 10 impinging droplets.
Li et al. [52]	Fluorinated DEG (FEP)	4 mA / 2000 V / 2.6 kW/m^2^	—	—	– Achieved several kW/m^2^ power density through replenishment of charged droplets via a Kelvin water dropper. – Demonstrated practical utility by lighting five 6‐W commercial lamps and charging a cellphone.
Wang et al. [67]	Fluorinated DEG (FEP)	0.28 mA / 525 V / 28.23 mW	—	—	– 3.5 times performance enhancement by employing high‐entropy ceramics as the intermediate layer of DEG. – Utilizing it as a biosensor to detect bacterial concentration.
Wu et al. [68]	Non‐fluorinated DEG (Natural leaf)	41 µA / 1.3 V / 1 µW /—	102°	—	– Developed a fully biodegradable DEG constructed entirely without plastic materials. – Evaluated five distinct leaf species to optimize and achieve high‐efficiency energy harvesting.
Wang et al. [44]	Non‐fluorinated DEG (Acrylic acid modified organic silicone resin)	450 µA / 140 V /—	94°	—	– Achieved an extended lifespan and cost reduction by utilizing silicon‐modified acrylic resin. – Demonstrated a large‐scale application with a surface area of up to 100 cm^2^.
Min et al. [45]	Non‐fluorinated DEG (PDMS with carnauba wax / tap water)	12 µA / 124 V / 1.2 W/m^2^	103°/11.5°/—	—	– Developed a DEG with a low sticky hydrophobic surface by embedding carnauba wax into PDMS. – Demonstrated its application as a water quality sensor for monitoring temperature, pH, and heavy metal ion concentrations.
Yu et al. [69]	Non‐fluorinated DEG (Palymitoylchloride + lignin coated cellulose paper)	0.47 µA / 4.7 V /—	163°/6.2°/5°	‐ Tensile strength of 50.2 MPa, fracture point at 4.8% strain	– Proposed fluorine‐free, petal‐inspired superhydrophobic interface fabricated from cellulose paper and esterified modified lignin. – Achieved high durability against 7‐day water immersion, abrasion, tape‐peeling, and folding.
Kamare et al. [46]	Non‐fluorinated DEG (Operculum beeswax)	20–40 µA / ∼500 V / 10–20 mW	106°	—	– Developed a sustainable and degradable DEG utilizing bio‐based waxes. – Demonstrated robust voltage recovery following cyclic humidity sweeps (35% to 95%) or temperature sweeps (30°C to 53°C).
Jang et al. [70]	Non‐fluorinated DEG (PDMS)	15 µA / 45 V / 42 µW	—	– Stretch rate of 170%, Young's modulus of 1.27 MPa – Over 99% in the wavelength range of 400−900 nm	– Proposed a hydrogel‐based DEG (Hy‐DEG) designed for seamless integration into everyday environments. – Incorporated the Hy‐DEG into a photovoltaic cell for a sun‐raindrop dual‐mode energy harvester.
Xu et al. [21]	Fluorinated liquid‐infused TENG (Krytox GPL103)	0.02 𝜇A / 1.2 V / 25 nW	—	– Over 60% in the wavelength of 600−1000 nm	– Introduced a slippery lubricant to enable TENG operation in high humidity and low temperatures. – Maintained stable output across a wide temperature range (−5°C to 55°C) and high humidity (∼90% RH).
Chen et al. [71]	Fluorinated/Non‐fluorinated liquid‐infused TENG (Mineral oil, silicone oil, PFPE oil)	3.5 𝜇A, 3.8 𝜇A, 3 𝜇A /—/ 5 𝜇W	121.3°/5°/‐	—	– Developed a slippery‐surface‐based TENG capable of all‐weather operation. – Proven self‐healing properties and stable power output in high humidity (∼90% RH).
Song et al. [72]	Fluorinated liquid‐infused DEG (Krytox GPL103)	5 mA / 100 V / 118 W/m^2^	—	—	– Proposed a lubricant‐armored DEG resilient to harsh conditions such as high salinity, caustic pH levels, high humidity, and low temperatures. – Utilized standard PCB technology to enable large‐scale integration.
Cui et al. [73]	Non‐fluorinated liquid‐infused TENG (Dimethyl silicone oil with silicone elastomer)	3 𝜇A / 5 V / 15 𝜇W	Over 90°	—	– Introduced an organogel‐based triboelectric layer to facilitate fast sliding speeds and large contact areas. – Demonstrated a 300% performance enhancement over PDMS‐based TENG.
Rajabi‐Abhari et al. [74]	Fluorinated liquid‐infused DEG (PFPE oil)	8.15 𝜇A / 1.1 V / 3.35 𝜇W	—/27°/—	– Over 80% in the wavelength of 550−700 nm	– Proposed a dual‐functional water and energy harvesting system utilizing patterned hydrophilic/hydrophobic surfaces. – Achieved stable performance for a 60‐day testing duration.
Yang et al. [75]	Non‐fluorinated non‐liquid slippery DEG (Ecoflex with erucamide microparticle)	9.5 𝜇A / 43.3 V / 21.8 𝜇W	105.9°	– Stretching rate of 300%, Young's modulus of 0.032 MPa, fracture point at 763%	– Proposed an improved durability TENG with efficient charge transfer enabled by dispersed lubricant particles. – Achieved stable performance for 1000 droplets and a 2‐month operation.
Soltani et al. [76]	Non‐fluorinated/fluorinated non‐liquid slippery TENG (2‐[methoxy (polyethyleneoxy)6‐9propyl]trimethoxysilane, 1,3‐dichlorotetramethyldisiloxane, PFPE)	0.002 𝜇A, 0.01 𝜇A, 0.021 𝜇A /—/—	—/3°/—	—	– Explored contact line dynamics in triboelectrification using liquid‐like polymer brushes. – Demonstrated an application as a pressure sensor.
This Work	Non‐fluorinated, non‐liquid slippery DEG (PDMS‐SCAL)	222.44 𝜇A / 162.2 V / 116.4 W/m^2^	108.6°/2.8°/4.2°	– Stable voltage during 1000 bending cycle with bending radius of 7 mm. – Over 80% transmittance in the wavelength of 470 to 900 nm	– Conducted comprehensive durability test, including mechanical (bending, abrasion, pencil hardness) and chemical durability (immersing into pH 2, pH 12, and 3.5 wt% NaCl for 24 h) – Maintained stable performance through 10 000 droplets, 3 months of laboratory storage, and 1‐month outdoor exposure (10 precipitation events and temperatures ranging from 24°C to −17°C). – Demonstrated reliability through field testing in rainy environments.

Although the PDMS‐SCAL‐DEG has demonstrated superior performance and long‐term stability, further characterization and optimization are needed before use in real applications. First, despite the developed PDMS‐SCAL‐DEG demonstrating excellent energy harvesting performance with fluorine‐free materials, its performance is still lower than that of DEGs using fluorine‐based materials [8]. Many studies have been conducted to implement SCAL surfaces using various polymer chains such as polyethylene oxide [77], perfluorinated polyether [78], and perfluoroalkane [79]. However, it is still necessary to develop SCAL coating materials that exhibit both excellent triboelectric energy harvesting performance and are fluorine‐free. In addition to changing the polymer chemistry, molecular‐level control of the PDMS‐SCAL brush architecture may provide another route for optimizing DEG performance. Parameters such as grafting density, chain length, and terminal group identity can be an effective strategy for further optimizing droplet retention and contact‐line dynamics in DEG operation [80, 81], and we believe it could provide useful guidance for future research. Additionally, many studies have been conducted to improve the energy harvesting performance of DEG platforms through various methods (novel materials [82, 83], DEG platforms [84, 85, 86], and hybrid energy harvesting systems [87, 88, 89]). Research verifying their performance in practical applications for industrial or consumer devices is still limited. Furthermore, exposure to outdoor environments can also lead to harsh chemical conditions, including variations in pH, humidity, and UV exposure. Therefore, in addition to limited analysis of energy harvesting performance characteristics in laboratory environments, verification of device performance under long‐term comprehensive operating conditions in the real world is necessary.

## 3 Conclusions

In this study, we developed a droplet‐based electricity generator (DEG) platform using a PDMS‐SCAL coating to overcome the limitations of superhydrophobic‐DEGs. In the case of a superhydrophobic‐DEG, the energy harvesting performance is significantly reduced, even with slight deviation from the optimal droplet release point, due to droplet bouncing and splitting. The PDMS‐SCAL‐DEG significantly reduces droplet rebound and splitting while maintaining effective droplet removal, resulting in stable energy harvesting performance. Unlike superhydrophobic surfaces that require precise droplet release locations for optimal output, the PDMS‐SCAL‐DEG exhibits drop‐location insensitivity, maintaining ∼75% and ∼50% of its peak performance even with 14.1 mm and 21.2 mm deviations from the optimal drop‐location, respectively. Additionally, the PDMS‐SCAL‐DEG shows optical transparency (>80% transmittance in the visible‐NIR range) and mechanical flexibility (no significant degradation throughout 1000 bending cycles at a bending radius of 7 mm), enabling potential integration into various applications. Through field testing in rainy environments, the reliability of PDMS‐SCAL‐DEG was also verified. This work demonstrates a previously unexplored PDMS‐SCAL‐DEG platform enhanced to broaden the applicability of energy harvesting devices.

## 4 Materials and Methods

### 4.1 Fabrication of Superhydrophobic‐ and PDMS‐SCAL‐DEG Platforms

Superhydrophobic‐ and PDMS‐SCAL‐DEG surfaces were fabricated to compare energy harvesting performance. Commercial ITO‐PET‐PE (100 Ω, MSE PRO Flexible Conductive Film Sheet for Solar Cells, MSE Supplies) films were used as substrates (width of 50 mm and length of 70 mm). First, the superhydrophobic coating solution was prepared by mixing 1 wt% Teflon amorphous fluororesin (CAS no. 37626‐13‐4, AF2400, Chemours) in a fluorinated solvent (CAS no. 86508‐42‐1, Fluorinert FC‐40, Sigma‐Aldrich) with 0.1 g/ml of PTFE nanoparticles (PTFE NPs, CAS no. 9002‐84‐0, Microdispersion‐200, Polysciences). The mixture was then stirred at 800 rpm for 1 h in a closed container. The superhydrophobic‐DEG surface was prepared by pouring the prepared solution onto the PE side of the ITO‐PET‐PE substrate, followed by spin‐coating (Spin‐1200T, Midas system) at 500 rpm for 1 min and curing at 165°C for 15 min.

To realize the PDMS‐SCAL coating on the ITO‐PET‐PE substrate, an SiO_2_ layer and a PDMS‐SCAL coating layer were deposited using the layer‐by‐layer method as shown in Figure S18. The SiO_2_ layer exhibits a high affinity with the silane coupling agent and stable surface hydroxyl groups [90, 91], which allows the PDMS brush of the PDMS‐SCAL solution to bond to the surface covalently. For this, sputtering (AJA ORION 3 sputter system, AJA International) was used as an SiO_2_ source to deposit a thickness of 100 nm on the PE side of the ITO‐PET‐PE substrate with a deposition rate of 0.2 Å/s at RF power of 200 W and base chamber pressure of 1.5 × 10^−6^ Torr (Figure S18a). The PDMS‐SCAL was coated by dip‐coating (Figure S18b), with the SiO_2_‐coated samples dipped into the PDMS‐SCAL coating solution prepared according to the method of Wang and McCarthy [30], which uses sulfuric acid as a catalyst and water molecules in the air to “instantly” bond PDMS brushes on the surface. While the surface prepared according to the recipe by Wang and McCarthy is generally classified as a “slippery omniphobic covalently attached liquid (SOCAL)” coating. However, in this study, we have consistently used the term “PDMS‐SCAL” to emphasize that the surface consists of PDMS brushes covalently bonded, rather than focusing on the fabrication method itself. They explained that SOCAL is formed by acid‐catalyzed hydrolysis/condensation during the drying after substrate withdrawal from the IPA/DMDMS/H_2_SO_4_ solution, since the water molecules act as co‐monomers (Figure S18c). They observed that no polymerization occurred within the bulk solution for up to 12 h, which means the effect of variations in immersion time on PDMS brush polymerization is minimal. For this reason, they observed that the polymerization density decreased in low‐humidity environments, which can increase the CAH. They also mentioned that applying heat treatment during drying allows the fabrication of brushes with larger thickness by a rapid polymerization rate, but it leads to reduced uniformity and increased CAH, consequently.

The key process tolerance lies in the surface chemistry and uniformity of the underlayer rather than the coating conditions (except for the humidity). For example, Zhao et al. [26] reported that direct PDMS‐SCAL deposition can fail on metal surfaces, and a uniform SiO_2_ interlayer provides the hydroxyl‐rich chemistry and smooth interface necessary for PDMS‐SCAL bonding. For the same reason, we used a 100 nm SiO_2_ intermediate layer in this study to achieve successful covalent bonding of the PDMS‐SCAL coating. Specifically, the PDMS‐SCAL solution was prepared by mixing 100 g of isopropanol (CAS No. 67‐63‐0, Sigma Aldrich), 20 g of dimethoxydimethylsilane (DMDMS, CAS No. 1112‐39‐6, Sigma Aldrich), and 1 g of sulfuric acid (CAS No. 7664‐93‐9, Sigma Aldrich), respectively. The mixture was vigorously stirred using a magnetic stirrer and then used after a 30 min stabilization period. The SiO_2_‐deposited ITO‐PET‐PE surfaces were immersed in the PDMS‐SCAL solution for 30 s and then dried vertically at room temperature for 1 min. The dried surfaces were fabricated by removing the remaining PDMS‐SCAL solution using DI water and drying in a clean nitrogen (N_2_) flow. Although this study didn't cover it, Wang and McCarthy's ‘SOCAL’ route has been further optimized and systematically examined. Armstrong et al. [92] demonstrated reproducible low‐pinning SOCAL surfaces, and Barrio‐Zhang et al. [93] clarified CAH and contact line friction characteristics of these liquid‐like surfaces. Notably, Gresham et al. [81] summarize and compare various protocols for producing liquid‐like PDMS‐SCAL coatings and highlight how reaction variables and synthesis details influence the final SCAL properties. It should be noted that, because we used H_2_SO_4_ during the PDMS‐SCAL coating process, residual acid during the acid‐catalyzed surface fabrication process can affect surface properties and long‐term stability. Indeed, such concerns have been repeatedly raised in the previous literature. For instance, Takeuchi et al. [94] analyzed acid‐washed Ti surfaces using XPS and reported that sulfur‐containing residues could be detected after H_2_SO_4_ treatment, but there was no Cl ion detection after HCl treatment. Furthermore, the PDMS‐SCAL coating recipe from Wang and McCarthy [30], which was adopted in this study, includes a rinsing step to remove residual sulfuric acid. To directly verify this, we further measured the XPS S 2p region of the as‐prepared PDMS‐SCAL surface. As a result, we observed no significant S 2p peaks, as shown in Figure S19, which implies that no measurable levels of residual sulfuric acid/sulfate species remain on the surface.

### 4.2 Characterization of Morphology, Wettability, and Optical Transmittance

The morphologies of the fabricated DEG surfaces (both superhydrophobic and PDMS‐SCAL coating) were investigated using an SEM (JSM‐7000F, JEOL). The cross‐section of the DEG samples was prepared using a focused ion beam (FIB) milling machine (Scios, FEI) for thickness analysis. For the quantitative analysis of sample thickness, measurements were averaged across 3 samples, with 3 measurements per sample. An AFM (Asylum Research Cypher, Oxford Instruments) was used for quantifying the surface roughness. After scanning a 5 µm × 5 µm area, the built‐in planefit algorithm with the first order XY‐direction was used to correct skew. The roughness parameter is chosen as the root mean square roughness value (*R*_q_). For the quantitative analysis of surface roughness, measurements were averaged across 3 samples, with 3 measurements per sample. For the wettability analysis, a contact angle goniometer (Model 250, Ramé‐Hart Instrument Co.) was used with a 10 µL water droplet volume. Wettability measurements were averaged across five samples, with 10 measurements per sample. It should be noted that some uncertainty may have arisen in the measurements of CA and CAH. This can arise from various experimental‐ and environmental‐related factors. Specifically, measurements can be influenced by experimental factors [28, 81, 95], such as the precise control of droplet volume (or contact line velocity), vibration, and stage tilt, or environmental factors [96], such as evaporation and humidity. The transmittance of the DEG was measured at wavelengths from 300 to 900 nm by a UV–vis‐NIR spectrophotometer (Cary 5000, Agilent).

### 4.3 Characterization of Energy Harvesting Performance

The open‐circuit voltage output of all DEGs was characterized by an oscilloscope (DSOX3024T, Keysight) connected with a passive probe (CT4026, Cal Test Electronics). The upper electrode was locally positioned on the dielectric surface. Specifically, it had a diameter of 0.35 mm and a length of at least 7 cm to cover the full width of the sample and the electrode connection. It was aligned parallel to the x‐direction of the device. The short‐circuit current output was characterized by the oscilloscope connected to a current preamplifier (SR 570, Stanford Research Systems) with a sensitivity of 100 𝜇A/V. All the DEGs were attached to a stage (Model 384, NRC) to adjust their inclination angle. Droplets were generated by an intravenous filled with DI water (CAS no. 7732‐18‐5, Aqua Solutions, Inc.), and the droplet height (*h*) was controlled by adjusting the outlet of the intravenous. After measuring the voltage of all DEGs, the current was calculated according to Ohm's law, considering the input resistance of 200 MΩ of the passive probe. The energy output (*E*_out_) was calculated from the integration of the voltage output (*V*)‐ operation time (*t*) graph as follows:

(1) $$E_{out}=\int_{0}^{T}\frac{V^{2}}{R}dt$$

where *T* and *R* represent the time at which the DEG operation is completed and the input resistance of the passive probe, respectively. The power density (*P*) was calculated using the peak voltage (*V* ^2^_peak_) across the resistors and the droplet maximum contact area (*A*, *A* = 2.49 cm^2^ for PDMS‐SCAL‐DEG at *h* = 25 cm, *θ* = 45°) as follows:

(2) $$P=\frac{V_{peak}^{2}}{R\times A}$$

To quantify the uniformity of open‐circuit voltage output according to droplet impact conditions, the *CV* for each condition was derived as follows:

(3) $$CV=\frac{\sigma }{Avg}\times 100\%$$

The average (*Avg*) and standard deviation (*σ*) were calculated using ten peaks for each condition. LEDs (NSPG500DS, Nichia) and a capacitor (1 MFD 50V monolithic ceramic capacitor, IOFIT) were prepared to demonstrate electrical energy outputs. The amount of capacitor charge was analyzed by measuring the voltage every 2 s using a multimeter (179 True RMS Digital Multimeter, Fluke). It is important to protect the ITO electrode from unwanted contact, particularly with water, which can significantly degrade energy harvesting performance by short‐circuiting. So we used Kapton tape to protect the ITO electrode and carbon tape as the contact electrode to measure the performance of DEG (both PDMS‐SCAL and superhydrophobic). For *CV* analysis, 3 measurements were performed on each of the 3 samples, and the results were averaged. All electrical peak analyses were averaged over 10 peaks.

### 4.4 Characterization of Comprehensive Durability

The cyclic bending test was conducted using a home‐built bending tester, referring to the previous report [97]. Specifically, to fix the bending radius for the cyclic bending test, we measured the change in electrical resistance of the ITO film as a function of bending radius. During the ITO film bending test, the ITO‐coated surface faces upward, thereby inducing tensile stress. Similarly, during the PDMS‐SCAL‐DEG film‐bending test, the PDMS‐SCAL‐coated surface faces upward. Coating hardness was evaluated by using a pencil hardness test according to ASTM D3363. Specifically, the pencil was positioned at a 45° relative to the surface, and hardness was measured using a series of pencils ranging from 9B to 9H under a load of 500 g. Abrasion tests were conducted using an automatic cyclic abrasion tester (Model 5900 Reciprocating Abraser, Taber Industries), referring to the previous report [98]. Specifically, the tests were performed under a vertical load of 1 N (31.6 kPa) at a horizontal reciprocating speed of 10 cycles per min. To evaluate the chemical durability, samples were immersed in H_2_SO_4_ (pH 2), NaOH (pH 12), and 3.5 wt% NaCl (simulating seawater) aqueous solutions for 24 h, respectively, and the open‐circuit peak voltage was compared. To prevent corrosion of the ITO film, all samples were protected on the backside (ITO side) with Kapton tape before immersion. Furthermore, because short‐circuiting caused by ionic liquid permeation can significantly compromise energy harvesting performance, the DEG performance was measured after the samples were fully dried for more than 1 day.

## Conflicts of Interest

The authors declare no conflicts of interest.

## Supporting information




**Supporting File 1**: advs76947‐sup‐0001‐SuppMat.docx.

**Supporting File 2**: advs76947‐sup‐0002‐MovieS1.mp4.

**Supporting File 3**: advs76947‐sup‐0003‐MovieS2.mp4.

**Supporting File 4**: advs76947‐sup‐0004‐MovieS3.mp4.

**Supporting File 5**: advs76947‐sup‐0005‐MovieS4.mp4.

**Supporting File 6**: advs76947‐sup‐0006‐MovieS5.mp4.

**Supporting File 7**: advs76947‐sup‐0007‐MovieS6.mp4.

## Data Availability

The data that support the findings of this study are available from the corresponding author upon reasonable request.
