# Supplementary material for: Drop‐Location‐Insensitive Droplet‐Based Electricity Generator Using Polydimethylsiloxane Slippery Covalently Attached Liquid Coatings
Source: Adv Sci (Weinh). 2026 Aug 5;13(60):e76947. doi: 10.1002/advs.76947 (PMC13440203; doi:10.1002/advs.76947)
Supplement: Supplementary file 1 [file ADVS-13-e76947-s006.docx]

**Supporting Information**

**for**

Drop-Location-Insensitive Droplet-based Electricity Generator Using Polydimethylsiloxane Slippery Covalently Attached Liquid Coatings.

*Jung Bin Yang ^a,b,†^, Donghyeon Yoo ^a,c,†^, Hideaki Teshima ^a,c,d^, Donghan Lee ^e^, Dongwhi Choi ^e^, Dong Rip Kim ^b,*^, Nenad Miljkovic ^a,c,f,g,h,i,*^*

^a^ Department of Mechanical Science and Engineering, University of Illinois at Urbana-Champaign, Urbana, IL, USA

^b^ School of Mechanical Engineering, Hanyang University, Seoul, 04763, South Korea

^c^ International Institute for Carbon-Neutral Energy Research (WPI-I2CNER), Kyushu University, Motooka 744, Nishi-ku, Fukuoka 819-0395, Japan

^d^ Department of Aeronautics and Astronautics, Kyushu University, 744 Motooka, Nishi-ku, Fukuoka 819-0395, Japan

^e^ Department of Mechanical Engineering (Integrated Engineering Program), Kyung Hee University, Yongin, Gyeonggi 17104, Republic of Korea

^f^ Materials Research Laboratory, University of Illinois at Urbana-Champaign, Urbana, IL, USA

^g^ Department of Electrical and Computer Engineering, University of Illinois at Urbana-Champaign, Urbana, IL, USA

^h^ Institute for Sustainability, Energy and Environment (iSEE), University of Illinois, Urbana, IL, USA

^i^ Air Conditioning and Refrigeration Center, University of Illinois, Urbana, IL, USA

* Corresponding author.

E-mail: D. R. Kim: [dongrip@hanyang.ac.kr](mailto:dongrip@hanyang.ac.kr) and N. Miljkovic: [nmiljkov@illinois.edu](mailto:nmiljkov@illinois.edu)

†These authors contributed equally to this work as co-first authors.

**Figure S1.** Cross-sectional tilted-view scanning electron microscope (SEM) images of commercial ITO-PET-PE substrate. Platinum (Pt) was used as a focused ion beam (FIB) protection layer.

**Figure S2. Surface characterization and energy harvesting performance of the superhydrophobic-droplet-based electricity generator (DEG) surface.** (a) Top-view scanning electron microscope (SEM), (b) atomic force microscope (AFM), (c) and cross-sectional tilted-view SEM images of superhydrophobic-DEG surface. (d) Water static contact angles of superhydrophobic-DEG surface. (e) X-ray photoelectron spectroscopy (XPS) survey spectra of the superhydrophobic-DEG surface and its elemental quantifications. (f) Open circuit voltage output of the superhydrophobic-DEG surface during continuous water droplet impact. (g) Open circuit voltage output and (h) energy output (*E*_out_) on the superhydrophobic-DEG generated by a single water droplet impact. The droplets were dropped from a droplet release height, *h* = 25 cm and an inclination angle of the sample, *θ* = 45°. DI water is used with a volume of ~ 49 µL in all droplet impact experiments.

**Figure S3. Schematic illustration of the energy harvesting mechanism and equivalent circuit of DEG.** When a droplet approaches DEG, the dielectric layer of DEG is negatively charged after sequential contact and separation with previous droplets. (ii) After droplet impacts and spreads along the surface, dipoles and ions of the droplet are arranged, and positive charges in the electrodes move to make a new electrostatic equilibrium. During DEG operation and equivalent circuit, three capacitors and a resistor constitute a closed circuit. In this circuit, *C*_EDL,1_ represents a capacitor of liquid droplet-dielectric interface, and *C*_Bulk_ represents a capacitor of liquid droplet-dielectric-ITO, and *C*_EDL,2_ represents a capacitor of liquid droplet-upper electrode interface. RD is primarily determined by the resistivity of the liquid droplet. (iii) When the droplet starts to shrink, the direction of voltage output is reversed. (iv) One cycle for energy generation of DEG is completed when the droplet completely detaches from the DEG.

**Figure S4. Surface potential measurement according to the existence of residual droplets.** Surface potential results of polydimethylsiloxane (PDMS) slippery covalently attached liquid (SCAL) DEG: Initial, after 100 droplets impinging, and after residual droplets.

**Figure S5.** **Surface characterization of PDMS-SCAL-DEG surface.** (a) Top-view SEM, (b) AFM, (c) and cross-sectional tilted-view SEM images of PDMS-SCAL-DEG surface. (d) Water static contact angles of PDMS-SCAL-DEG surface. (e) XPS survey spectra of the PDMS-SCAL-DEG surface and its elemental quantifications. (f) Open circuit voltage output of the PDMS-SCAL-DEG surface during continuous water droplet impact. (g) Open circuit voltage output and (h) energy output (*E*_out_) on the PDMS-SCAL-DEG generated by a single water droplet impact. The droplets were dropped from *h* = 25 cm and *θ* = 45°. DI water is used with a volume of ~ 49 µL in all droplet impact experiments.

**Figure S6. AFM force-curve analysis for measuring the PDMS-SCAL brush thickness.** Representative approach and retract force curves measured on the PDMS-SCAL surface. The inset schematic image stands for the state of the AFM tip corresponding to the force curve.

**Figure S7.** Si 2p peaks measurement by XPS analysis of (a) SiO_2_ layer and (b) SiO_2_-PDMS-SCAL layer. The red and green areas indicate the fitted peak of O-Si-O bond and O-Si(CH_3_)_2_-O bond.

**Figure S8. Solid-solid contact electrification test results.** (a) Photograph of the pushing test setup. Triboelectric property results from measuring the solid-solid contact electrification experiment of (b) the superhydrophobic- and (c) the PDMS-SCAL-DEG platform.

**Figure S9. Open-circuit output peak voltage according to the thickness of the SiO_2_ intermediate layer of PDMS-SCAL-DEG platforms.** Error bars represent the standard deviation of the total 6 independent measurements on the two samples in each SiO_2_ thickness.

**Figure S10.** **Visualization of the small droplet-sized (~ 15 µL) water droplet behavior on the superhydrophobic-DEG surface using a high-speed camera.** The droplet height is 25 cm, inclined angle is 45°.

**Figure S11. Current and charge distribution according to the y-direction displacement.** (a) Short circuit current and (b) transferred charge of the Superhydrophobic- and PDMS-SCAL-DEG according to the y-direction displacement. The droplet height was 25 cm, and the inclined angle was 45°. DI water is used with a volume of ~ 49 µL in all droplet impact experiments.

**Figure S12. Droplet impact dynamics on PDMS-SCAL- and superhydrophobic-DEG at various y-direction displacement from the optimal point.** (a) Contact area variation over time for PDMS-SCAL-DEG. (b) Side views of maximum rebound height before departure from the superhydrophobic-DEG. (c) Top views of droplet impact showing the maximum splitting before leaving the superhydrophobic DEG surface. *h* and *θ* were 25 cm and 45°, respectively. DI water is used with a volume of ~ 49 µL. *y* is the y-directional displacement from the point where the DEG performance is maximum.

**Figure S13. Peak analysis of the positive and negative parts of the open-circuit output voltage.** (a) Voltage output of the PDMS-SCAL-DEG with a 56 MΩ external resistance. (b) The red and blue shaded areas indicate the harvested energy components by the positive and negative voltage.

**Figure S1~~4~~. Open-circuit output peak voltage of superhydrophobic-DEG according to the droplet volume.**

**Figure S15. Charging performance of superhydrophobic-DEG.** The droplet height was 25 cm and an inclined angle was 45°. *y* is the y-directional displacement from the point where the DEG performance is maximum.

**Figure S16. Bending test results of ITO film.** (a) Resistance changes of ITO film according to the bending radius. Δ*R* = *R*-*R*_0_, and *R* is measured value of resistance and *R*_0_ is initially measured resistance. (b) SEM images after bending test with bending radius of 5 mm for ITO and PDMS-SCAL surface. Error bars in (e) represent the standard deviation of the total 3 independent measurements on the one sample in each bending radius.

**Figure S17. Surface morphology of PDMS-SCAL-DEG after a 1-month outdoor exposure test.** Top-view, cross-sectional tilted view SEM, and AFM images of PDMS-SCAL-DEG after a 1-month outdoor exposure test.

**Figure S18. Schematic diagram of the fabrication process of the PDMS-SCAL-DEG platform.** PDMS-SCAL-DEGs were realized by a 2-step fabrication process: (a) SiO_2_ sputtering process and (b) dip coating. (c) Details of the PDMS-SCAL coating process. During the PDMS-SCAL coating process, dimethyldimethoxysilane is hydrolyzed, and a grafting reaction occurs via H_2_SO_4_ catalysis.

**Figure S19. S 2p peaks measurement by XPS analysis of the PDMS-SCAL-DEG.** No significant S 2p peaks were observed.
